# Targeting the Tumor Microenvironment: A Close Up of Tumor-Associated Macrophages and Neutrophils

**DOI:** 10.3389/fonc.2022.871513

**Published:** 2022-05-19

**Authors:** Massimo Russo, Claudia Nastasi

**Affiliations:** ^1^ Laboratory of Cancer Metastasis Therapeutics, Department of Oncology, Mario Negri Pharmacological Research Institute (IRCCS), Milan, Italy; ^2^ Laboratory of Cancer Pharmacology, Department of Oncology, Mario Negri Pharmacological Research Institute (IRCCS), Milan, Italy

**Keywords:** tumor-microenvironment, macrophages, neutrophils, anti-cancer therapy, TAMs, TANs, cancer

## Abstract

The importance of the tumor microenvironment (TME) in dynamically regulating cancer progression and influencing the therapeutic outcome is widely accepted and appreciated. Several therapeutic strategies to modify or modulate the TME, like angiogenesis or immune checkpoint inhibitors, showed clinical efficacy and received approval from regulatory authorities. Within recent decades, new promising strategies targeting myeloid cells have been implemented in preclinical cancer models. The predominance of specific cell phenotypes in the TME has been attributed to pro- or anti-tumoral. Hence, their modulation can, in turn, alter the responses to standard-of-care treatments, making them more or less effective. Here, we summarize and discuss the current knowledge and the correlated challenges about the tumor-associated macrophages and neutrophils targeting strategies, current treatments, and future developments.

## 1 Introduction

Cancer is a significant cause of death worldwide and does so through the ability of malignant cells to egress from the primary mass and spread to other parts of the body *via* a complex process known as metastasis. This latter can be seen as secondary cancer, as it can profoundly differ from the primary and progressively overwhelm organs leading to death.

Resistance to cancer treatment can be intrinsic to the tumor cells, but it is often conferred by non-malignant ones that make up the tumor microenvironment (TME). The importance of the TME stands within its capacity to dynamically regulate cancer progression and influence the response to treatment. For this reason, several therapies target different components of the TME, aiming to shatter at least one pillar of the palace.

The TME is considered a complex and rich multicellular environment where a tumor takes roots. It does not just include tumor cells but also many normal ones that can contribute both positively and negatively. Indeed, they can be modified by malignant cells and induced to synthesize growth factors, chemokines, matrix-degrading enzymes to enhance proliferation and invasion. They can also rearrange the stroma, avoiding the effective delivery of anti-cancer drugs, increasing interstitial fluid pressure, and changes in vascular flow (1, 2).

**Figure 1:**
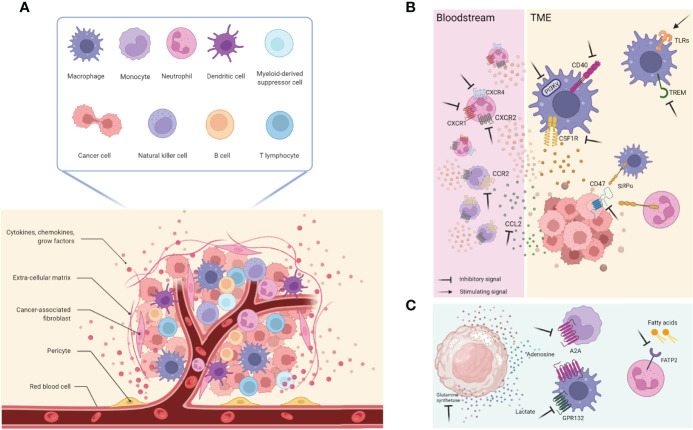
**(A)** The TME is composed by several diverse cell types including cancer cells, immune cells (such as T and B lymphocytes, macrophages, DCs, NK cells, MDSCs, neutrophils), and stromal cells (like pericytes, mesenchymal stromal cells, fibroblasts); this architecture is also supported by the extracellular matrix and its proteins, as well as growth factors and cytokines produced by all the cellular component that, in turn, influence the TME. Together with them, blood and lymphatic vascular networks allow exchanges and nutritional supply. **(B)** General overview of cellular and molecular targets currently used and on development. **(C)** New relevant targets involving cancer cells metabolites and receptors expressed by immune cells. This figure was made with Biorender.

Many are the cell types involved: immune cells, such as dendritic cells (DCs), macrophages, T and B lymphocytes, natural killers (NKs), neutrophils, myeloid-derived suppressor cells (MDSC); stromal cells like pericytes, mesenchymal cells, cancer-associated fibroblasts (CAFs); the extracellular matrix (ECM) with many secreted molecules as cytokines, chemokines, growth factors; and the blood and lymphatic vascular networks, which are in communication with each other and the tumoral cells (**Figure 1A**).

In the light of knowledge, we understand the early days of the TME research field where therapeutics targeting each component were viewed with enormous interest (3); now, we are aware of the TME complexity, and those early perspectives were seen as overly optimistic.

Such complexity resides within several different aspects like the stage of cancer, the organ in which the tumor arises, the ontogeny of some cell populations, and their phenotype within the tumor mass and/or at the systemic level.

More recently, the knowledge of the functional role of myeloid cells (macrophages, neutrophils, MDSC) and their interactions with tumor cells has remarkably increased. The types and the relative abundance of tumor-infiltrating leukocytes define the immune landscape, and it has been shown to have prognostic value. As the increase of cytotoxic CD8+ T cells is positively correlated with survival and treatment response, the presence of myeloid cells, depending on their phenotype, could be either negative or positive (4–6). Several studies have highlighted the correlation of a specific subset of myeloid cells with more prolonged survival and better clinical responses, showing myeloid cells’ heterogeneity in tumors.

The TME is well-recognized in regulating the response to therapeutic interventions conferring an intrinsic resistance or acquiring one.

High numbers of immunosuppressive cells, including tumor-associated macrophages (TAMs) and regulatory T cells (Treg), and the presence of protective niches that shield a subset of tumor cells from therapeutic effects, additionally contribute to intrinsic resistance (7–9). Few studies have revealed the pleiotropic adaptive effects on TME composition and phenotypes following different therapeutic interventions, including standard-of-care treatments and TME-directed therapies (7). These alterations lead to massive therapy-induced cell death and the correlated accumulation of immune cells, which phenotypes could be specific to the therapeutic intervention.

Both radiotherapy and chemotherapies can increase the presence of immunosuppressive TAMs in tumors, protecting the cancer cells from therapy-induced cell death, which may ultimately lead to tumor recurrence (10–13). For example, chemotherapy may also induce DNA damage in stromal cells, resulting in the activation of NF-κB contributing to therapeutic resistance (14), and radiotherapy may affect the tumor vasculature promoting cancer cell survival and radio-resistance (15); as well, specific therapies can show a synergistic effect by promoting immunogenic cell death and enhancing T cell-dependent anti-tumor immunity (16, 17).

Given the importance of the TME in tumor progression and the efficacy of cancer treatment, in recent years, the TME has been taken as a central focus for new therapeutic strategies. These approaches mainly target TAMs, neutrophils, DCs, T cells, tumor vasculature, ECM, and CAFs. This review will focus on the myeloid cell neutrophils and macrophages, their controversial role in TME, and the clinically relevant therapeutics that are currently in use or underway.

## 2 Myeloid Cells in Tumors

Since Rudolf Virchow recognized the immune system as an essential regulator of tumor growth, describing extensive accumulations of white cells within tumors (18), the presence of myeloid cells within the TME has raised substantial interest. Our comprehension of myeloid cells’ functionality and their interaction with tumor cells has given us an epiphany in the last decades.

Great endeavours to boost T cell-directed anti-cancer immune responses have been made to date. As reported, the incidence of cancerogenesis is low in invertebrates with no T or B cells, indicating that innate immune cells are of great importance for preventing the initiation and development of cancer (19, 20).

Since the study of the TME immunophenotype had been introduced and often paired with classical oncology screenings, pathologists and oncologists had to realize the predictive value of the immune landscape based on the evidence that specific cell types are associated with distinct disease outcomes in patients. Consequently, several immune-oncology strategies have been developed to reactivate the adaptive and innate immune systems to mount a proper immune response as an alternative approach to classical anti-cancer treatments.

The opening of new clinical trials using immune checkpoint inhibitors (ICIs) (such as monoclonal antibodies (mAbs) against cytotoxic T lymphocyte antigen 4 (CTLA4), programmed cell death 1 (PD1), and PD1 ligand (PDL1), have shown complete success only on a small fraction of patients with melanoma and lung cancers, and possible reasons are still unknown (21).

In preclinical studies, TMAs can promote the recovery of tumors despite chemotherapies, radiotherapies, or biologic therapies due to the promotion of angiogenesis, maintenance of cancer stem cells, and inhibition of immune responses (22, 23). In some tumors, macrophage infiltration also interferes with the efficacy of immunotherapy, neutralizing efforts to reactivate CD8+ T cells. For this reason, several therapeutic strategies to modulate TAM function, infiltration, or activation are emerging to block resistance to conventional therapies and promote T cell-based therapies (4, 22, 24).

In parallel, recent findings studying neutrophils in cancer have opened a debate about their involvement in tumor formation, progression, and dissemination, showing a dichotomy of their role. Moreover, the importance of neutrophil recruitment in tumoral tissues was assessed on human cancer samples. It was associated with a more aggressive disease characterized by inadequate treatment response, tumor relapse, and bad prognosis (25).

## 3 Neutrophils

### 3.1 About Neutrophils

Neutrophils are bone marrow (BM) granulocytic-myeloid cells and comprise 50-70% in humans and 10-30% in mice of circulating white blood cells, making the granulocytic population the first most abundant in humans and the second one in mice (B cells precede them) (26, 27). Historically, neutrophils are short-lived leukocytes that last about one day in the circulation and then cleared away by macrophages or dendritic cells in the liver, spleen, and BM (28). The granulocyte-colony stimulating factor (G-CSF) stimulates the proliferation and differentiation of neutrophil precursors into the BM and augments the mobilization of terminally differentiated neutrophils into the bloodstream (29). In fact, it regulates granulopoiesis, the *de novo* generation of neutrophils, both at the steady-state and at emergency. The latter occurs when an inflammatory stimuli (i.e. microbial infections or cancer) becomes systemic and considerably enhances the generation and the release of immature and mature neutrophils from the BM into the peripheral blood (30, 31). Alternatively, stress conditions (i.e. extensive blood loss, cancer, BM dysfunction) induce the extramedullary emergency hematopoiesis in the spleen that produces myeloid cells, monocytes, and neutrophils (32). Neutrophils developmental stages relate to their systemic trafficking. It is possible to distinguish fully mature neutrophils from pre-neutrophils and immature neutrophils using the expression of the CXC-chemokine receptor 2 (CXCR2) found only on the surface of fully differentiated neutrophils (29, 33). CXCR2 mediates signaling required for neutrophil mobilization into the peripheral blood by interacting with the IL-8 [a.k.a. Neutrophil chemotactic factor or CXC-chemokine ligand 8 (CXCL8)]. IL-8, released by endothelial cells and stromal cells of the basement membrane, acts as a chemoattractant to recruit circulating neutrophils to the site of inflammation and is required to mediate the rolling of neutrophils along the endothelium (34). Conversely to CXCR2, the CXC-chemokine receptor 4 (CXCR4) is expressed on immature neutrophils (both proliferating and mitotically inactive), where it mediates the signaling required for neutrophils retention into the BM compartment (29).

The Ly6G (Lymphocyte antigen 6G) is the key marker of neutrophils, but it is not only present on fully mature and mitotically inactive neutrophils, but also on precursors, as recently proved by 10X technology (35). Hence, it should not be considered to discriminate between immature and mature neutrophils. The distinction between those ones further relies on the gene signature level; for example, the Gata1 gene is more expressed by BM mature neutrophils than in their precursors; on the contrary, the Gata2 gene is highly expressed in premature rather than mature neutrophils (35). Nowadays, transcriptomic advances and multiparametric flow cytometry analyses revealed the presence of several neutrophil subsets both in mice and humans. The pre-neutrophil (preNeu), a committed proliferative precursor, differentiates into mitotically inactive immature (immNeu) and mature neutrophils (31). Analogously, the presence of an early neutrophil progenitor (NeP) in mouse BM and a similar unipotent NeP in human BM (hNeP) were identified using cytometry by time of flight (CyTOF). NeP was further classified into two clusters, C1 and C2 (based on Ly6G marker), giving rise exclusively to neutrophils (35). These findings have been very recently confirmed, exploiting single-cell RNA sequencing (RNAseq), partitioned differentiating and mature subtypes of neutrophils into eight clusters (G0-G4, G5a-G5c) (33). Neutrophils are the first responders to danger signals (sterile insults or microbial infections) and are among the first mediators of inflammatory reactions. Their fast migration is mediated by danger-associated molecular patterns (DAMPs), pathogen-associated molecular patterns (PAMPs), and the activation of the Toll-like receptors (TLRs). At the injury site, they can release the content of their cytoplasmic granules or exert their protective roles by the respiratory burst producing reactive oxygen species (ROS), extruding the Neutrophil Extracellular Traps (NETs) (36), or by acting as antigen-presenting cells (APC) (37, 38).

### 3.2 The Controversial Role of Neutrophils in Cancer

Nowadays, the knowledge about neutrophils is constantly expanding. These cells are not only circulating grenades but also mixed populations capable of adapting and specializing in micro-environmental cues (39); thus, they exert both pro and anti-cancer activities. Notably, tumor-associated neutrophils, both primaries or secondaries, are usually referred to as TANs, even though this terminology does not relate to a specific differentiation step and activation status (40). Pertinent to metastases, the role of neutrophils is quite confounding and closely resembles the case of primary cancers. Neutrophils have been described to elicit both metastasis-promoting and -suppressing capacities, depending on the cancer type, staging, and micro-environmental signals or cancer cell-intrinsic causes. Different findings suggest their direct or indirect involvement in mediating an anti-cancer immune response early during carcinogenesis. In preclinical models, neutrophils were shown to delay primary tumor growth by releasing nitric oxide (NO) that induces cancer cell killing. The binding of the receptor tyrosine kinase MET, expressed on neutrophils, by the tumor necrosis factor-alpha (TNFα), and its ligand, the Hepatocyte growth factor (HGF), drives the nitric oxide synthase (NOS) and the consequent release of the anti-tumor inflammatory mediator (41). The neutrophil elastase (ELANE) is another extracellular protein released by human polymorphonuclear cells that can directly kill cancer cells. While human neutrophils release a catalytically active ELANE, instead murine neutrophils do not and hence fail to kill cancer cells, both *in vivo* and *in vitro* (42). A study, carried out with a mouse model spontaneously developing epithelial carcinogenesis due to the functional loss of the tumor suppressor phosphatase and tensin homolog (PTEN), neutrophils were shown to exert an inhibitory role in developing endometrial adenocarcinoma by inducing the detachment of tumor cells from the basement membrane (43), implying that PMNs can fight autochthonous tumorigenesis.

The involvement of the chemokine receptor CXCR2 in skin and intestine tumors development has been assessed when its deficiency or depletion (on Ly6G^+^ cells) suppressed colitis-associated tumorigenesis and the formation of intestine adenomas (44). Nonetheless, the lack of a mouse model with neutrophil knockout for CXCR2 made the findings controversial. However, mouse models with selective deletion of CXCR2 in neutrophils could be generated by crossing MRP8Cre, and Cxcr2fl/fl mice (45).

PMNs are part of immune cell networks that suppress tumor formation and growth by activating T cell-mediated anti-tumor responses. During the early phase of the development of a murine sarcoma induced by 3-methylcholanthrene, neutrophils amplified the production of IL12 by macrophages, which in turn drove the release of the interferon-gamma (IFNγ) by a subset of unconventional T cells, establishing an anti-tumor immunity that led to a reduced incidence of sarcoma (46). In early-stage human lung cancer, immature neutrophils, influenced by the low concentration of INF-γ and GM-CSF in the TME, differentiated into hybrid TANs with an APC phenotype, cross-presenting tumor antigens to T cells, in turn stimulating their response and unleashing their anti-tumor action (47).

Earlier it was shown that granulocytes are equipped with anti-metastatic functions. In fact, in mice orthotopically transplanted with murine breast cancer cells 4T1, was demonstrated that tumor cells recruited neutrophils, *via* CC-motif chemokine ligand 2 (CCL2), into the pre-metastatic lungs; once arrived, neutrophils produced hydrogen peroxide (H_2_O_2_) that prevented the seeding of disseminated tumor cells (48). Another line of evidence reported that in human renal cancer, neutrophils were actively recruited to the metastasis sites, thanks to tumor-derived IL8 and CXCL5, exerting an anti-metastatic action (49). In these studies, neutrophils were recruited and instructed to dampen metastasis formation by a tumor-released soluble signal representing an example of tumor entrained neutrophils (TEN). Furthermore, another study revealed that MET is required for neutrophil chemo-attraction and cytotoxicity in response to HGF. Consequently, the release of HGF/MET-dependent NO by neutrophils promotes cancer cell killing, abating metastasis formation, and corresponding primary tumor growth (in several cancer models) (41).

As well, the genetic inactivation of the atypical chemokine receptors 2 (ACKR2), a scavenger for inflammatory chemokines and hence a negative regulator of inflammation (expressed in hematopoietic precursors), resulted in the release from the BM neutrophils showing higher anti-metastatic activity in mice orthotopically transplanted with 4T1 mammary carcinoma or intravenously injected with B16F10 melanoma (50).

Conversely, others demonstrated the involvement of neutrophils in the tumor formation or the growth of established primary tumors. Neutrophils might support tumor formation, incidence, and growth by exploiting several mechanisms, including the promotion of a chronic inflammatory state which was extensively studied and reviewed by others (25, 40, 51), inducing DNA damage and genome instability (52) or inducing the proangiogenic switch (53).

In the context of chemically induced tumorigenesis, PMNs amplify the DNA damage caused by urethane, a component of cigarette smoke, in murine lungs, stimulating cancer initiation (54). Similarly, the release of ROS by neutrophils was shown to trigger oxidative DNA damage mutations causative of intestinal tumorigenesis and consequent cancer growth (55).

Neo-angiogenesis is the sprouting and growth of blood vessels into a tumor mass promoted by tumor cells and secreted growth factors and cytokines, useful for the oxygen and nutrients supply required for tumor growth (56). Primary mediators of cancer-induced neo-angiogenesis are the vascular endothelial growth factors (VEGFs) that include VEGF-A, VEGF-B, VEGF-C, VEGF-D, and placental growth factor (PLGF) and their respective receptors, the vascular endothelial growth factor receptors (VEGFRs) and the neuropilin (NRPs). Neutrophils are a reservoir of pre-formed VEGF, although they are not the principal producers of the growth factor as in the case of macrophages (57). Still, neutrophils release VEGF and other angiogenic factors such as the protein Bombina variegate peptide 8/Prokinecitin 2 (Bv8/PROK2), contributing to alternative pathways leading to new blood vessels formation (58, 59). On the contrary, neutrophils are the primary source of metalloproteinase-9 (MMP-9), which degrades the ECM and forces mesenchymal cells to release VEGF-A and other proangiogenic molecules such as the fibroblast growth factor (FGF) (60).

Another mechanisms has revealed that neutrophils, by IL1-β and matrix MMP, inhibit NK cells cytotoxicity and the disseminated tumor cells (DTCs) extravasation into the target organ, promoting their survival (61). As proof, in immunocompetent or NOD-*scid* mice, bearing E0071 breast carcinoma, neutrophils showed metastasis supporting activity *via* NK suppression, while in NSG mice (lacking of NKs), they reduced metastasis (62).

Additionally, neutrophils secrete pro-inflammatory factors (i.e., lipids and cysteinyl leukotrienes) able to favor metastatic initiating cells MICs, leading to increased metastatic competence of breast cancer cells like 4T1 (63).

It was also reported that IL-17, secreted by γδ-T cells, induces neutrophil accumulation in the lungs which, in turn, suppresses cytotoxic T cells functions, increasing pulmonary and lymph node metastasization in the KEP mouse model of spontaneous breast cancer metastasis (64). Here neutrophil depletion caused a reduction of pulmonary metastasis formation.

Interestingly, in the bloodstream has been reported an interaction between circulating tumor cells (CTCs) and white blood cells, predominantly with neutrophils. This connection seems to drive CTC cycle progression and the induction of CTCs metastatic potential (65). Such peculiar interaction could be seen a potential new target for targeting for treatment of metastatic breast cancer.

Proteomic approaches elucidated the TANs secretome, identifying transferrin as the major mitogen for tumor cells. Depletion of neutrophils inhibited lung metastasis and transferrin production in the metastatic microenvironment, while deletion of transferrin receptors suppressed the growth of lung-colonizing tumor cells (66). By these findings, preclinical models of tumors different from the mammary ones [like lung (67) and colorectal cancer (68)] produce neutrophils that support metastasis. Furthermore, others reported a pathological Notch signaling involvement in colorectal cancer cells that initiates a neutrophil-dependent, and tumor-necrosis factor (TGF)- β mediated signaling cascade leading to the appearance of metastatic disease (68).

Nonetheless, different other stimuli affect PMN cells, modulating their role in metastasis. For example, the loss of testosterone in castrated male mice impaired neutrophils’ maturation and functions, thereby making them pro-metastatic in two preclinical models, while testosterone replacement restored their cytotoxic functions. These results were also observed in patients with prostate cancer undergoing androgen deprivation treatments (69).

Besides being a potent anti-tumor protein, ELASTASE is also one of the primary markers used to detect NETosis (NETs formation). NETs are extracellular, web-like structures composed of decondensed nuclear and mitochondrial DNA intertwined with cytotoxic enzymes, such as neutrophil elastase (NE) and myeloperoxidase (MPO), that usually are retained into neutrophil granules and used to neutralize pathogens like bacteria, fungi, viruses, and parasites (70). NETs formation is a mechanism that cancer cells adapt to hijack neutrophils to awake disseminated dormant cancer cells (71) or to enhance the establishment of metastases in triple-negative breast cancer murine models (72). Such a link is not only confined to hematogenous metastases but was also observed in a preclinical model of ovarian cancer, where the metastatic dissemination occurs through a transcelomatic process. By the way, it seems that the neutrophils influx into the omentum is a prerequisite for a successful metastatic dissemination. In detail, ovarian tumor-derived inflammatory factors stimulate neutrophils to NET which, in turn, binds ovarian cancer cells and promotes metastasis. In fact, omental metastasis is decreased in mice with neutrophil-specific deficiency of peptidyl-arginine deiminase 4 (PAD4), an essential enzyme for NET formation (73). Similarly, neutrophils have been shown to induce hepatocellular carcinoma (HCC) metastasis by releasing NETs that, once internalized by HCC cells, activate the TLR4 and subsequently induce cell-death resistance and enhanced invasiveness (74).

### 3.3 Neutrophils Interference With Anti-Cancer Therapies

Given the extensive and growing body of evidences related to the involvement of neutrophils in the formation and progression of cancer, it is relevant to understand whether they might affect anti-cancer treatments.

Immunotherapy is the latest breakthrough in anti-tumor therapy and is used to make the immune system reactive against cancers taking advantage of the blockade of immune checkpoints. In the context of cancer, immunosuppressive determinants present in the TME downregulate the immune cells’ reactivity, making them exhausted or polarised toward a pro-tumor profile (75).

Antibodies against a different number of ICIs that support the expansion of type-I helper CD4^+^ T lymphocytes and prevent the exhaustion of CD8^+^ T cells have reached the clinic (76). Unfortunately, not all patients respond to ICIs, possibly owing to intrinsic resistance, but many usually develop acquired resistance (77).

In this context, neutrophils or, more specifically, TANs are considered contributors to the resistance to ICIs. In hot tumors (highly infiltrated with T lymphocytes), the ICIs are usually effective unless the TME is enriched with TANs, suggesting a granulocytic immunosuppressive role (78). The neutrophil pathological activation by the microenvironmental stimuli exerts detrimental effects on T cells and thus mediates the resistance to ICIs. Different neutrophil mediators are able to induce T cells exhaustion, including arginase 1, prostaglandin E2, ROS, and NO, as recently reviewed elsewhere (79).

A therapeutic strategy to restore the sensitivity to ICIs is to dampen neutrophils recruitment in the TME, hence avoiding their hijacking (80). The receptors CXCR2 and CXCR4 are central regulators of neutrophil trafficking and recruitment in tumors. In fact, the inhibition of CXCR2 in a murine model of a soft tissue sarcoma resistant to anti-PD1 treatment restored the effectiveness of the immunotherapy (81). It was also reported that the treatment with a small-molecule inhibitor of CXCR2 and CXCR1, SX-682 (SX), sensitized tumor-bearing mice to the anti-PD1 antibody. The inhibitor had no anti-tumor effect in monotherapy and was ineffective on cancer cells, independently of their positivity for CXCR1 and CXCR2 (82). These preclinical findings provided a rationale for a clinical translation, thus that ongoing clinical trials are evaluating the effectiveness of combining CXCR2 inhibitors with ICIs. SX is currently being investigated in combination with pembrolizumab and with nivolumab (both targeting PD1) for the treatment, respectively, of metastatic melanoma [NCT03161431 (83)] and metastatic pancreatic ductal adenocarcinoma [PDAC; NCT04477343 (84)]) and unresectable or metastatic colorectal cancer [NCT04599140 (85)].

Like CXCR2 inhibition, targeting CXCR4 represents a different strategy to reduce tumor recruitment and neutrophil mobilization from the BM. Accordingly, treatment with AMD3100 (plerixafor, Mozobil), a small-molecule inhibitor of CXCR4, promotes an enhanced intratumoral immune B and T cell responses in metastatic lesions in patients with microsatellite stable (MSS) colorectal cancer (CRC) or pancreatic ductal adenocarcinoma (PDA), that usually are ICIs resistant (86). Concomitantly, the clinical trial NCT02907099 (87) is studying the inhibitor of CXCR4, BL-8040, in patients with metastatic PDAC.

Pulmonary endothelial cells express CXCL12 constitutively. Treatments with AMD3100 was shown to cause neutrophilia (neutrophils in the bloodstream), decrease of neutrophils in the BM, and induce neutrophil distribution in the lungs without compromising their trafficking to inflamed sites (88). Conversely, the release of neutrophils from lungs into the bloodstream was previously observed during the treatment with AMD3100 (89). When considering the inhibition of CXCR4 in lung cancer, it needs to be considered the immunosuppressive neutrophil recruitment in the metastatic lesion and hence reduced response to ICIs.

Considering that neutrophils recruited into the tumor may acquire a tumor-supporting phenotype, the inhibition of their recruitment could be associated to the modulation of their phenotype. To this aim, a preclinical trial, on mice bearing the allografts 4T1 and LLC or transplanted with the triple-negative breast cancer line MDA-MB-231, showed that the CXCR2-inhibitor SX in combination with BinTrafusp Alpha, that simultaneously blocks PD-L1 and traps soluble TGF-β, polarize neutrophils (90, 91). SX-682 and BinTrafusp Alpha are being tested in co-administration in phase I/II trials on solid metastatic cancers (NCT04574583) (92).

A possible therapeutic strategy to induce anti-tumor neutrophils in cancer and potentiate ICIs is to potentiate the appearance of anti-tumor neutrophils, like with INF-γ. It was shown that early treatment with anti-PD1 Ab induced tumor shrinkage in mouse models of pancreatic cancer. On the contrary, delayed anti-PD1 treatment showed limited benefits associated with CXCR2^+^ myeloid cell recruitment in response to tumor secreted CXCL8. The administration of INF-γ inhibited the tumor trafficking of CXCR2^+^ cells, suppressing the release of tumor-derived CXCL8, ultimately enhancing anti-PD1 efficacy (93). This combination is currently investigated in a clinical trial for advanced solid tumors [NCT02614456 (94)];, which results have not been deposited yet. A general representation of cellular and molecular targets are visualized in **Figure 1B**.

Angiogenesis Inhibitors (AIs) block the formation of new blood vessels into the tumor and have been investigated in monotherapy for renal cancer and, more often, in combination with conventional chemotherapy. The VEGF/VEGFR, the angiopoietins (ANGPT) and their tyrosine kinase receptors (Tie2/Tek), or molecules mediating tumor angiogenesis, like the fibroblast growth factor (bFGF/FGF2) and platelet-derived growth factor-B (PDGF-B), have been the main targets. The humanized anti-VEGF monoclonal Ab bevacizumab, the VEGF-Trap protein aflibercept, and small molecules inhibitors of VEGF-receptors (i.e. sunitinib, sorafenib, pazopanib, and cediranib) represent examples of currently US Food and Drug Administration (FDA)-approved antiangiogenic drugs (56). The use of AIs was promising in preclinical studies but not effective in the clinical setting.

To this extent, neutrophils have been observed to sustain the resistance to therapy by generating of alternative vascularisation mechanisms. For example, tumor-infiltrating neutrophils produced the Bv8/PROK2 protein, which caused the refractoriness to anti-VEGF therapy in tumor allograft and xenograft models (95). The same was observed in a genetically engineered mouse model (GEMM), spontaneously developing colorectal cancer (CRC), and in mice bearing the colon cancer cells CT26 and Colon38. In these models, therapy resistance occurred only upon the induction of an inflammatory state by chemically-induced colitis, which caused the augmentation of the G-CSF serum levels in mice, followed by the recruitment of neutrophils into the tumor stroma and the release of Bv8/PROK2, promoting angiogenesis. Treatment with anti-GCSF or anti- Bv8/PROK2 rescued the anti-VEGF tumor sensitivity (96). These findings indicate that the tumor responses to AI may rely on the degree of inflammation of tumor tissues.

Another study correlated the degree of neutrophils infiltrated into the metastasis with the bevacizumab treatment refractoriness and the decreased overall survival (OS), both in xenograft and syngeneic tumor models. Furthermore, the depletion of neutrophils or the use with BI-880, which targeted a different angiogenic pathway (the Tie2/Tek axis), restored the sensitivity to anti-angiogenic treatment (97). Additionally, in a preclinical model of glioblastoma, neutrophils were again found to support anti-VEGF therapy resistance in mice (98).

In contrast with these preclinical findings, the high absolute neutrophils count and high neutrophils to lymphocyte ratio (NLR) have been considered prognostic factors for antiangiogenic treatment efficacy and favorable prognosis in CRC patients, respectively (99, 100). However, the opposite is also supported in other studies (101).

Neutrophils might also indirectly promote resistance to antiangiogenic therapy and hence tumor progression. It was highlighted that TANs, but not circulating neutrophils, *via* the production of CCL2 and CCL17 and the recruitment of monocytes and Treg cells, were the cause of the refractoriness to sorafenib, the first line treatment for hepatocellular carcinomas (HCC) (102, 103). These studies do not elucidate or demonstrate whether neutrophils or the pathologically activated PMN-MDSCs were the one responsible for the acquisition of such resistance. It would be of great interest to investigate the characteristics of the neutrophil population in cancer patients undergoing anti-cancer treatments by the newly available molecular biology techniques such as RNAseq to verify each specific subset’s contribution to therapy response.

Despite novel therapies, conventional cytotoxic-based chemotherapy remains the cornerstone in cancer treatment. Consequently, in preclinical and clinical trials, the effectiveness of new agents is frequently investigated with conventional chemotherapy. Neutrophils have emerged as contributors to cancer progression, reducing tumor responsiveness to the chemotherapy rather than mediating resistance. Therefore, chemotherapy might delay tumor growth, failing to induce its shrinkage. Thus, the rationale behind the co-administration of chemotherapy and immunomodulators acting on neutrophils is again to reduce their recruitment in the tumor and a likely pro-tumor polarization or convert immunosuppressive neutrophils (PMN-MDSCs) toward an anti-tumor phenotype. For example, one preclinical evidence highlighted the effectiveness of combining cisplatin, a widely known chemotherapeutic drug, with the inhibition of CXCR2 axis (104); the authors showed that the agent SB225002, that selectively inhibits CXCR2, enhanced the therapeutic effect of cisplatin in lung cancer mice models, which was associated with a significant reduction of neutrophil infiltration and enhanced CD8+ T cell anti-tumor. Supporting the benefits of combinatorial treatment, in preclinical models of PDAC, it has been tested FOLFIRINOX (composed of oxaliplatin, irinotecan, 5-FU, and leucovorin) with the small molecule PF-04136309, a CCR2 inhibitor for limiting the recruitment of monocytes-macrophages (105). This combination resulted in compensatory recruitment into the tumor of neutrophil populations highly expressing CXCR2. The combined CCR2 plus CXCR2 blockade enhanced the chemotherapeutic efficacy and improved the survival of tumor-bearing mice. Moreover, inhibiting the CXCR2 axis with the molecule SB 225002 in combination with paclitaxel retarded the growth of Lewis Lung Carcinoma (LLC) bearing mice (106). These findings are very recent, and currently, no clinical trials are using CXCR2 inhibitors with chemotherapy.

Conversely, agents exerting an immunomodulatory effect, like INF-γ, upon neutrophils have been explored. Several clinical trials are still recruiting to investigate the benefits of adding INF-γ to chemotherapy, principally phase I/II trials where the main objective is to determine the best-tolerated dose. Given the low number of patients recruited, the estimation of response parameters is considered secondary outcomes.

Patients undergoing chemotherapy frequently develop neutropenia when the absolute neutrophil count (ANC) drops below 2 X10^9^/L. The Grade 4 neutropenia (<0.5 X10^9^/L) represents a life-threatening event requiring patient hospitalization, treatments with antibiotics (to avoid the risk of spreading infections), and chemotherapy discontinuation, possibly favoring tumor relapse. The drop in the ANC that occurs during neutropenia usually persists for 2-3 weeks, possibly leading to a reduced abundance of neutrophils within the tumor bulk, making their targeting not feasible. The hematopoietic grow factor G-CSF (Filgrastim) is an FDA-approved drug for patients with non-myeloid malignancies, used to reduce the time for neutrophil recovery and to decrease the incidence of infections. The Granulocyte macrophage-colony Stimulating Factor (GM-CSF or Molgrastim) might represent another hematopoietic growth factor used as an immune-stimulant agent to treat neutropenic patients. A clinical trial shows that Molgrastin induced a faster neutrophil recovery and reduced hospitalization but the drug worked in a limited number of patients compared to Filgrastim, thus it is considered less effective than G-CSF. In another trial conducted on non-small-cell-lung-cancer patients, the GM-CSF was ineffective.

Alternatively, it could be hypothesized that neutrophils could be targeted based on the administered chemotherapy, since each treatment differentially affects neutrophils (e.g., cyclophosphamide and doxorubicin are drugs more myelotoxic than 5-fluorouracil or methotrexate) (107).

Chemotherapy-induced neutropenia may be considered *per se* an approach to target neutrophils by depleting their precursors in the BM. Intriguingly, neutropenia is associated with drug effectiveness and better overall survival (108), although it may cause therapy discontinuation or delayed treatment cycles. However, it is still unknown if chemotherapy-induced neutropenia and the reported outcome improvements are linked or unrelated events, and prospective trials designed *ad hoc* to evaluate this association is still lacking.

### 3.4 Current Strategies to Target Tumor-Associated Neutrophils

The above-mentioned strategies aim to increase the effectiveness and efficacy of the current standard of care treatments. Still, they do not represent *per se* a strategy that exploits the neutrophils’ anti-cancer killing machinery, as in the case of autologous and heterologous T-cell-based therapies recognizing specific tumor antigens and mediating a direct cancer cell killing (109). Even if neutrophils are ontogenically endowed with anti-cancer properties, a tumor-promoting phenotype is more frequently observed and defined as a tumor-induced conversion subset referred to as G-MDSC. In light of this, it would be clinically relevant to find a druggable target that favor neutrophil reprogramming toward their naturally occurring anti-tumor phenotype. To this regard, the fatty acid transport protein 2 (FATP2) was recently identified as a regulator of the suppressive capacities of G-MDSCs; it is a neutrophil membrane protein implicated in the trafficking of lipids and it is overexpressed by G-MDSCs in tumor-bearing mice compared to “classical” neutrophil isolated in healthy mice. The pharmacological inhibition of FATP2 by lipofermata resulted in a delay in tumor growth of mice bearing different and etiologically unrelated tumors (namely: LLC and EL4), without affecting the proliferation of the same tumors cultured *in vitro* (**Figure 1C**). It should be noted that the *in vivo* anti-tumor effect was lost when mice were treated with lipofermata and an anti-CD8 depleting antibody or when the therapy was administered in immunodeficient (NOD/SCID) non-obese diabetic–severe mice, indicating that the anti-tumor effect is anyway mediated by T cells rather than neutrophils (110). Of note, this finding highlighted the role of the immune cells’ metabolism and the influence on their polarization.

Neutrophils often share signaling cascades and extracellular receptors with other myeloid cells, such as monocytes. Using a strategy not explicitly tailored towards neutrophils might paradoxically exert detrimental effects on other myeloid cells. Hence, a more profound comprehension of neutrophils’ intricate roles in tumor progression might provide new ideas for new therapeutic approaches. Despite this scary scenario, the innate immune checkpoint SIRPα/CD47, a negative regulator of myeloid cell phagocytosis, is a druggable axis which impairment showed efficacy at the preclinical levels, even though it is not a neutrophil specific target but rather a complex shared with monocyte and macrophages. For example, breast tumor-bearing mice benefitted from the combinatorial treatment with the mAb trastuzumab directed against the human epidermal receptor 2 (HER2) and the blockade of the SIRPα/CD47 checkpoint interaction that increased the killing activity of neutrophils towards antibody-opsonized cancer cells and led to tumor shrinkage caused by the antibody-dependent cell cytotoxicity (ADCC) (111) (**Figure 1C**).

As already described, several reports showed the opposite effect of neutrophils in different steps of tumor progression. Contrasting effects on metastasis formation were observed depending on the type of studied tumor. For example, after neutrophils depletion (anti-Ly6G mediated), a drastic reduction of secondary tumors (49) and a critical increment of metastatic deposits (63) were reported in Renca and 4T1 breast cancer bearing mice, respectively. This observation supports the notion of a cancer-induced alteration of the neutrophil functions. More interestingly, it suggests that comparing differences and analogies between different cancers, achieved *via* omics-based methods, might reveal new pathways involved in the pathological activation of neutrophils.

## 4 Macrophages

### 4.1 About Macrophages

Macrophages, a type of white blood cell deriving from a myeloid progenitor, play essential roles in maintaining tissue homeostasis and protecting our body through several functions, such as engulfing and digesting foreign substances. Macrophages also clear away harmful matter, including cellular debris and tumor cells *in vivo*, maintaining homeostasis and limiting the entrance of pathogens (112–114).

These cells are classically categorized in the innate compartment of the immune system since they mediate a non-specific response and help initiate a specific defense mechanism, typical of adaptive immunity. In addition to stimulating the immune system, macrophages exert an immune-modulatory impact by secreting various cytokines and activating the complement system, leading to inflammation.

Depending on the microenvironment, chemokines, cytokines, and other stimuli (local anoxia, lactic acid), these cells can shape their phenotype. This led to the pragmatic description 15 years ago of two divergent forms of macrophage activation, such as M1 with immunostimulatory and anti-tumoral activity, and M2, immunosuppressive, both linked to the arms of the adaptive immune system with which they interact (T helper cells). This fundamental dichotomy has essentially formed the basis of research into macrophage activity ever since (115). Evidence indicates that macrophage phenotypes may be more appropriately described as a continuum of functional states that are signal-dependent and plastic (116), making it even more complex to classify cancers based on TME myeloid composition.

Macrophages have long been hypothesized to originate from cells of the blood compartment, deriving from hematopoietic BM precursors that would be attracted and recruited at peripheral tissues upon inflammatory conditions or tissue damage (112–114).

The understanding of macrophages ontogeny has recently undergone a profound transformation thanks to modern lineage tracing techniques. The main notable discovery is that most tissue-resident macrophages are not derived from BM progenitors, as previously thought, but instead from the yolk sac or fetal liver (117, 118). In adults, some tissue-specific macrophages exclusively derive from one source (for instance those in the intestine derive from BM). In comparison, in other tissues (i.e. the skin), different batches of macrophages derive from one source or another. Within the brain, ontogenetically distinct macrophage populations exist, including both tissue-resident microglia and BMDMs (119). The first develops from embryonic yolk sac progenitor cells and are not replenished postnatally through peripheral mononuclear haematopoiesis (120). Also, non-parenchymal macrophages within the CNS arise during embryonic development, and are largely stable populations during adult life (121). By contrast, in response to perturbations of tissue homeostasis or pathological conditions, circulating monocytes are recruited to the brain parenchyma and give rise to BMDMs through monocytosis, particularly during tumor progression where the integrity of the blood-brain-barrier (BBB) is compromised (122).

Furthermore, the yolk sac-derived macrophages of the heart are replaced by fetal liver monocytes (118, 123). The presence of persistent embryonic populations throughout life in most tissues suggests that these tissues harbor pre-macrophages (pMACs) that can proliferate to give rise to mature macrophages (123).

Decades of shreds of evidence support that the environmental niche strongly influences macrophage gene expression and function, yet these cells remain plastic and retain the capacity to alter their phenotypes in response to new signals and situations. Phenotype is ultimately a flexible translation of multilevel cell-intrinsic and environmental signals.

Like healthy tissues, tumors also contain diverse populations of signal-responsive macrophages. Local mediators and conditions may significantly influence macrophage polarization in the tumor context as tumors have an evolving and chronic pathology that may involve dynamic environmental stresses such as hypoxia (124). Circulating precursors that are recruited into tumor tissues and subsequently differentiate into TAMs include conventional inflammatory monocytes and monocyte-related myeloid-derived suppressor cells (M−MDSCs). This latter can differentiate into mature TAMs when the signal transducer and activator of transcription 3 (STAT3) are downregulated (125); additionally, M-MDSCs contribute to the immunosuppressive tumor microenvironment, promoting tumor metastasis (126).

Of note, TAM proliferation has been observed in mouse models of sarcoma and mouse and human breast carcinomas, but this general mechanism does not seem to sustain the numbers of TAMs in growing tumors, suggesting that recruitment of circulating cells is required to maintain the TAM population (127, 128).

In a tumor setting, M1-like macrophages are currently thought to promote anti-tumor immunity, whereas M2-like TAMs stimulate angiogenesis and tissue repair (127) and suppress cytotoxic T cell function indirectly promoting tumor progression. In reality, heterogeneous macrophage populations coexist within the tumor compartment, influencing the progression of both tumor growth and the evolving immune response (124, 129). Nonetheless, a full understanding of the heterogeneity and functional states of TAMs seem now more relevant in clinical and therapeutic settings than ever before, as recently supported by the collected evidences (130).

A considerable number of questions have been raised about the relevance of macrophages’ phenotype according to their lineage compared with their tissue environment, whether the replacement of yolk sac-derived or fetal liver-derived macrophages with BM-derived macrophages results in identical phenotypes, and whether macrophages from different origins can be specifically targeted. In the context of tumors, these questions are essential because not all TAMs take origin from the same organ (119, 131). For example, in pancreatic cancer models, the yolk sac-derived macrophages show a pro-tumoral phenotype opposite to the BM-derived macrophages, suggesting that origin is important (132).

Such observations underline that a different origin might be of clinical relevance, and they raise questions as to whether inhibition of BM-derived macrophage recruitment might result in compensation by yolk sac-derived and/or fetal liver-derived tissue progenitors or vice versa. Above the yet complex scenario of cancer, it seems of great importance the understand macrophage origin, heterogeneity, and dynamics in the tumor microenvironment.

### 4.2 The Controversial Role of Macrophages in Cancer

Macrophages can potentially mount a robust anti-tumoral response, as they can directly eliminate cancer cells if adequately activated. They can also support the adaptive immune response by presenting tumor antigens and producing chemokines and cytokines to recruit and activate cytotoxic CD8+ T cells (CTLs) and NK cells.

In the 1970s, studies demonstrated that macrophages activated by bacterial products and cytokines acquire the capacity to kill tumor cells (133–136). At least for the initial stage of cancer, TAMs have been seen as an ally, whereas when tumors progress, the TME modifies the environment and the TAMs, supporting tumor progression. It had been found that TAMs from malignant metastatic cancers promote tumor growth and metastasis (134).

Thus, early evidence suggested that macrophages could engage in a controversial dual relationship with cancer. The tumor-promoting functions of TAMs are diverse and may impact the different stages of tumor progression, from cancer initiation to metastasis, contributing to different hallmarks of cancer. Macrophages have bimodal roles in orchestrating immune responses that can either hamper or foster the effectiveness of conventional anti-cancer strategies.

In the first stages of tumor formation, macrophages are mainly tumoricidal, as they show an activated state, producing reactive oxygen and nitrogen intermediates that may contribute to DNA damage and genetic instability (55). The role of macrophages in the transition from a benign to a malignant tumor has been studied in only a few cancer models (mammary, skin, and pancreatic cancers) (137–139) and, at least in mammary tumors, premature recruitment of macrophages by overexpression of colony stimulator factor 1 (CSF1) promotes the transition to malignancy (138).

Furthermore, the presence of type II cytokines (interleukin-(IL)-4 or IL-13) in the microenvironment affect macrophage functions and phenotypes resembling those involved in tissue development and repair, with consequent suppression of anti-tumoral response switching the immune response from a cytotoxic to a supportive role (24, 140).

Macrophages also exacerbate the transition to malignancy by producing angiogenic factors, proteases, and secretion of growth factors such as epidermal growth factor, which induces cancer cell proliferation and the support of epithelial-mesenchymal transition in tumor cells (141).

TAMs are a source of additional angiogenic factors, chemokines with proangiogenic and pro-lymphangiogenic potential, and inflammatory cytokines, including placental growth factor, fibroblast growth factor 2, VEGF-C, tumor necrosis factor (TNF), IL-1β, IL-6, and chemokine (C-X-C motif) ligand 8 (CXCL8) (53). Moreover, myeloid cells produce different proteases, such as MMPs and cathepsins, that mobilize ECM-bound VEGF-A and other factors. Hypoxia, a major driver of angiogenesis in cancer tissues, induces the upregulation of hypoxia-inducible factor-1α (HIF-1α) expression and secretion of proangiogenic factors, such as VEGF-A (142). In addition, myeloid-derived VEGF-A is essential for the tumorigenic alteration of the vasculature. This alteration delays tumor progression as VEGF-A deficiency in TAMs was found to reduce angiogenesis and abnormalities in tumor vessels in mouse cancer models but to increase tumor growth (143). Accordingly, TAMs are also promoters of the neoangiogenic switch in tumors since their frequency correlates with the vascular density in preclinical tumor models and human tumors (144).

In mice, Ly6C+/CCR2+ cells, defined as inflammatory monocytes, have been shown to contribute to TAM accumulation and maintenance in a mouse mammary tumor model (145) and the establishment of pulmonary metastases derived from mouse or human breast cancer cells (146). In contrast, a protective role of patrolling monocytes, defined as Ly6C-/CX3CR1+, is shown by their inability to extravasate into tissues and differentiate into macrophages; despite that, they can rapidly accumulate within lung metastases and inhibit tumor cell seeding and growth in mouse models. Such anti-tumor functions include scavenging tumor debris, the recruitment and activation NK cells, and Th1 responses (147, 148).

M2-like macrophages can be found in the metastatic cell niche at more advanced stages, where they exert trophic functions while promoting tumor-initiating cell evasion of immune clearance (149, 150).

TAMs are major drivers of immunosuppression in the TME. Mediators released by tumor-infiltrating lymphocytes, such as Th2 cells and Treg cells (producing IL-4 and Il-10), and by tumor cells (IL-10, TGFβ, and PGE2) activate an immunosuppressive program in TAMs (151, 152). Macrophage-derived cytokines, such as IL-1, promote the recruitment and seeding of metastatic cancer cells at niche sites (146, 149). Additionally, myelomonocytic cells also promote metabolic starvation of T cells due to the activity of arginase and the production of amino acid metabolites by indoleamine 2,3-dioxygenase (IDO).

Furthermore, in mouse and human melanoma, IL-1 was shown to induce the upregulation of the expression of TET2, a DNA methylcytosine dioxygenase, which sustained the immunosuppressive functions of TAMs (153). Finally, TAMs express the ligands of checkpoint molecules, such as PD-L1, PD-L2, B7 ligands (154), and VISTA (155), which suppress adaptive T cell immune responses and promote Treg recruitment (5, 156).

In addition to primary tumors, macrophages can also assist in tumor survival and colonization at premetastatic lesions. It has been shown that macrophages are required for the early dissemination of breast cancer, and early disseminated macrophages contribute to late metastasis (157).

Macrophages promote the invasiveness and metastasis of tumor cells by expressing matrix metalloproteinases, cathepsin, urokinase, plasminogen activator, and matrix remodeling enzymes (dissolving the extracellular matrix to pave the path for a tumor cell to escape, as well as secrete IL-1ra enhancing tumor cell stemness and metastasis (158)). It has also been observed that pancreatic cancer cell-derived exosomes preferentially colocalize with macrophages in liver metastasis sites (159). Exosome-educated macrophages facilitate premetastatic niche formation *via* secretion of TGF-β (160). Additionally, exosomes produced by macrophages can transfer miRNA into cancer cells favoring metastasization in colorectal cancer and pancreatic ductal adenocarcinoma cells (160, 161). Other studies support the indispensable role of monocytes/macrophages recruited to

premetastatic niches in promoting circulating tumor cell survival and colonization in metastatic lesions (162). For instance, tumor cells of lung metastasis (derived from breast cancer) produce CCL2, recruiting and retaining monocytes/macrophages (163), likewise fibrocytes that prepare the premetastatic niche for melanoma cells by the exact mechanism (164). The inflammatory monocytes CCR2+Ly6C+, after differentiating into Ly6C− macrophages, accelerate tumor cell extravasation by generating VEGF (146).

Tissue-resident macrophages have also been demonstrated to promote or restrict metastasis. Alveolar macrophages promote hepatocellular carcinoma lung metastasis by producing the inflammatory leukotriene B4 (165) and suppressing the Th1 responses (166). Conversely, Kupffer cells engulf cancer cells to limit liver metastasis (167).

Interestingly, within the brain, evidences support that the majority of TAMs tend to be pro-tumorigenic and accumulate with higher tumor grade (168) and the dogma of a simple linear M1-M2 phenotypic balance has been disputed. Instead, many groups are focusing on defining activation and phenotype as a measure of functional diversity in brain cancers (124).

Indeed, studies in mice showed that phenotypic alteration of TAMs results in anti-tumor efficacy in glioblastoma (169, 170), whereas TAM depletion prevents brain metastasis outgrowth (171).

Lately, it has been leveraged a diverse panel of analyses to deeply interrogate the immune landscape of primary and metastatic brain cancers uncovering several pronounced differences between gliomas and brain metastasis. A significant shift in the ratio of microglia and monocytes-derived macrophages (MDMs) has been revealed between isocitrate dehydrogenase 1 and 2 (IDH) mut and IDH WT gliomas. Additionally, gliomas show an abundance of TAMs, whereas T cells were much fewer (particularly in IDH mut tumors) confirming the notion that gliomas are immunologically cold tumors. By contrast, brain metastasis seem to accumulate more lymphocytes and neutrophils, indicating that tumors that arise within the brain shape their TME differently than cancers that metastasize from extracranial sites (172).

### 4.3 Macrophages Interference With Anti-Cancer Therapies

In conventional chemotherapy and radiotherapy, macrophages have boosted or limited the therapeutic effect. **Chemotherapy** can affect macrophages’ functions modulating the cross-talk with the adaptive compartment, thus changing the entity of the immune responses and ultimately the therapy outcome. More than 30 years ago, an interaction was reported between the chemotherapeutic agent actinomycin D and human and murine monocytes/macrophages, afterward named “drug-dependent cellular cytotoxicity” (173). Another earlier study underlined how immunity could determine the efficacy of doxorubicin treatment (174). This latter can induce tumor cells to undergo immunogenic cell death as they express alarm signals that trigger adaptive immune responses; for instance, doxorubicin causes a massive release of ATP from tumor cells leading to the mononuclear phagocytes recruitment and their differentiation in antigen-presenting cells (APCs) (175).

Data from preclinical models suggest that myeloid cells can shift the balance of the role of immunity in the anti-tumor activity of selected chemotherapeutic agents (176), which can be leveraged to increase the efficacy of ICIs (177).

Trabectedin, a DNA-binding agent that causes DNA damage and cell-cycle arrest in tumor cells, which the EMA approves for the treatment of soft-tissue sarcomas and ovarian cancer, and by the FDA for sarcoma therapy, has shown a complex mechanism aside from the conventional ones; it indeed affects the transcription of selected genes including some that encode for inflammatory cytokines and chemokines, as well as angiogenic factors (178). The secondary and relevant effect brings delayed and prolonged responses after trabectedin treatments; thus, that cannot be explained only by the effect on cancer cells (179, 180). It has been found to activate programmed cell death (through caspase 8) *via* cell-surface receptors selectively in monocytes, inducing their apoptosis (179). Furthermore, patients with sarcoma treated with trabectedin showed a reduced TAM infiltration and decreased angiogenesis, supporting the hypothesis that the reduction of macrophages abundance is a key component of the anti-tumor activity of this drug.

However, macrophages rarely have a positive effect on responsiveness to chemotherapy. TAMs, when polarized in M2 or M2-like, can limit the effectiveness of cytotoxic agents like platinum-containing compounds, paclitaxel, and doxorubicin (10, 115, 181–183). In mouse tumor-transplantation models, M2-like macrophages, orchestrating tissue repair, were found to accumulate in perivascular areas of the tumor after chemotherapy and promoted tumor revascularization and relapse (184); recruitment of these cells was found to be CXCR4–CXCL12 dependent (184). The discrepancy of the TAMs’ role in mediating the response to doxorubicin is probably a consequence of different mouse tumor models and their immunogenicity (10, 174, 181–183, 185). Patients with lymphomas treated with doxorubicin-containing regimens mirror preclinical data showing higher TAM infiltration associated with a favorable prognosis (174, 186). Similarly to these clinical associations, drugs like doxorubicin, oxaliplatin, and cyclophosphamide enhance the effect of chemotherapy through the induced immunogenic cell death (ICD), which implies the release of “eat me” signals from tumor cells promoting phagocytic and antigen-presenting capabilities (175, 176, 187, 188).

Additionally, chemotherapy can directly modulate the macrophage phenotype, reprogramming TAMs into M1-like immunostimulant cells, an effect observed with gemcitabine in pancreatic cancer (189), as well as 5-fluorouracil in colorectal cancer (190) and docetaxel in a preclinical model of breast cancer (191). Two general mechanisms seem to be responsible for the antagonistic effects of TAMs on chemotherapy outcomes. In mouse models, chemotherapy-induced tissue damage has been demonstrated to trigger the recruitment of immunosuppressive myeloid cells or elicit a pro-tumorigenic type 17 T−helper (Th17)-cell-skewed immune response promoted by IL−1 (182).

Alternatively, TAMs have been reported to protect mouse cancer stem cells (CSCs) from cytotoxicity (183, 185). Preclinical models, however, document primarily negative effects of macrophages on the responsiveness to chemotherapy; the several mechanisms identified include orchestration of an immunosuppressive response, tissue repair-related functions, nourishment of tumor cells, and pro-metastatic activity (4, 192). Accordingly, depletion of TAMs with anti-CSF1/CSF1R (CSF1 receptor) antibodies enhanced chemosensitivity to a combinatorial chemotherapeutic approach in human breast cancer xenografts (193) and a genetic mammary tumor model (194). Additionally, CSF1 expression correlates with accumulation of CD8+ T cells and CD163+ TAMs in melanoma, and anti−PD1 and anti−CSF1R combination therapy induced regression of melanoma in preclinical studies (195). Moreover, a mechanistic leap in our understanding of macrophage-specific targeting of the CSF1/CSF1R axis has been achieved in murine models of pancreatic ductal adenocarcinoma (137, 196); in fact, PD1 and CTLA4 antagonists showed limited efficacy as single agents to restrain tumor growth, but in combination with CSF1R blockade potently elicited tumor regressions, even in larger established tumors, providing a rationale to fuel the subsequent efforts to translate CSF1/1R-specific and other tumor-associated macrophage modulating therapies into the clinic (196).

Macrophage infiltration was found to be associated with chemoresistance to 5-fluorouracil in colon cancer cell lines (197), and macrophage depletion increased responsiveness to paclitaxel (PTX) treatment in breast cancer (10). Not to forget that TAMs foster chemoresistance releasing growth factors protecting tissues from chemotherapy-induced damage (183, 198). Of note, paclitaxel and doxorubicin increase the ability of perivascular macrophages to promote tumor cell metastasis.

The effect of radiotherapy on myeloid cells can also have dual implications for patient outcomes. In mouse models, the influx of monocytes into tumors following radiotherapy drives a profibrotic tissue response and might promote tumor recurrence (192, 199). Conversely, in patients, tumor regression at sites distant from the irradiated lesions — known as the ‘abscopal’ effect (200) — could plausibly be explained by activation of host anti-cancer immunity. In a mouse model, neoadjuvant low-dose γ−irradiation was found to set macrophage functions to an anti-tumor modality characterized by a lack of both immunosuppressive and proangiogenic activity and the production of T−cell−attracting chemokines (201). Instead, a fractionated cumulative radiation dose regimen, similar to those during cancer treatment, induced a pro-inflammatory phenotype in macrophages *in vitro* but did not alter their ability to promote cancer invasion and cancer angiogenesis (202).

Moreover, seeking to evaluate the applicability of radioimmunotherapy in experimental breast-to-brain metastasis models, it was reported that the induced immune modulation led to an increase in cytotoxic T-cell numbers and prevented the induction of lymphocyte-mediated immune suppression. Overall, radio-immunotherapy significantly improved tumor control with prolonged median survival, however recurrent brain metastases showed accumulation of blood-borne PD-L1+ myeloid cells, indicating the establishment of an immune suppressive environment to counteract re-activated T-cell responses (203). Therefore, TAMs can either reduce or amplify the magnitude of the anti-tumor effect of radiotherapy depending on context and TME; overall, data suggest that macrophage targeting in combination with radiotherapy could be a potential therapeutic strategy to modulate the stroma and allow better tumor killing, although it is not a well-explored field.

Another important determinant for the efficacy of chemotherapy and immunotherapy has emerged to be the host-microbiota (204–208). Mouse tumor models have been shown an essential role of microbial components in priming myeloid cells for the antineoplastic efficacy of platinum combined with adjuvant CpG oligonucleotides (204). Similarly, the antineoplastic activity of anthracyclines is compromised in mice with genetic inactivation of the formyl peptide receptor 1 (FPR1), a sensor of microbial components and tissue damage that is expressed in myeloid cells (209). The loss−of−function of the *FPR1* allele has been associated with unfavorable survival in patients with breast carcinoma or colorectal cancer after adjuvant chemotherapy, as well as blocking the receptor function with cyclosporin H (CsH) was shown to reduce the efficacy of anti-cancer chemotherapy against carcinogen-induced breast cancer (209, 210). Once more, myeloid cells determine the role of immunity in the anti-tumor activity of selected chemotherapeutic agents (176), which can be exploited to increase the efficacy of ICIs (177). In mouse models, repolarization of macrophages has also been reported in the context of targeted therapy, such as treatment of KIT−positive gastrointestinal stromal tumors (GISTs) with imatinib (211) and treatment of hepatocellular carcinoma with sorafenib (212).

Strategies targeting VEGF signaling are part of the current therapeutic armamentarium in oncology.

VEGF is a potent attractant of monocytes, acting *via* VEGFR−1, and its expression is upregulated in metastasis-associated macrophages in mammary carcinoma models (213). Although VEGF is a well-known chemotactic for monocytes, it did not drive the accumulation of macrophages in this model.

Nonetheless, VEGF signaling activates the CSF-1 pathway in metastasis-associated macrophages, taking that they are a major source of angiogenic factors, including VEGF; their density is also correlated to increased vasculature (214). Interestingly, the resistance of tumors to current anti-VEGF therapies is frequently associated with high levels of myeloid-cell infiltration (215). For instance, a study with 24 enrolled patients showed that the use of bevacizumab (anti-VEGF therapy) resulted in a pronounced increase in the number of TAMs and M2 macrophages compared to paclitaxel–carboplatin alone (used as neoadjuvant chemotherapy) (216). Furthermore, macrophage infiltration into human glioblastomas, resistant to anti-VEGF therapy, is correlated with a poor prognosis, but a combinatorial therapy with anti-VEGF and anti-ANG2 (angiopoietin-2) was shown to reprogram TAMs from M2 into M1 phenotype with relevant anti-tumor activity (217). Similarly, a vascular-disrupting agent 5,6-dimethylxanthenone-4-acetic acid (DMXAA), initially developed for disrupting tumor vasculature, has also been shown to activate immunostimulatory functions of TAMs, which in turn orchestrate anti-tumor response of CD8+ T cells (218).

While conventional therapies primarily target cancer cells, more recent treatments, especially mAb-based targeted therapies and immunotherapies, rely more profoundly on myeloid cells’ engagement (5).

The immunotherapy field has had a rapid expansion, particularly with the discovery of ICIs. Myelomonocytic cells are a vital component of the immunosuppressive pathways targeted by ICIs and might, therefore, offer tools to predict or increase the activity of such treatments. They express PD-1 ligands PD−L1 and PD−L2, as well as the CTLA−4 ligands B7−1 (CD80) and B7−2 (CD86), and the related protein B7−H4. PD−L1 and PD−L2 are upregulated on the surface of macrophages in response to various stimuli, including cytokines and hypoxia (219, 220). TAMs present in a variety of human tumor types often expresses different levels of high levels of immune-checkpoint molecules (214). The presence of these molecules is a predictor of response to therapy, especially to ICIs (221, 222). In preclinical models, FcγR-expressing macrophages eliminated CTLA−4−positive, mAb-coated Treg cells from tumors *via* ADCC (223, 224), unleashing anti-tumor immunity. The ADCC mediated by TAMs was shown in a study where melanoma patients responders to ipilimumab (mAb anti-CTLA-4) had higher numbers of circulating CD16+ monocytes and macrophages at tumor sites and lower Treg cells (225). In general, macrophages contribute to the TME immunosuppression through several mechanisms; thus, targeting TAMs might support the efficacy of ICIs by removing inhibitory factors for T cells (5). Up to date, neutralizing antibodies are currently US FDA-approved for the treatment of several cancers, including melanoma, advanced renal carcinoma, gastric cancer, non-small-cell lung cancer, and colorectal cancer; currently, they are also studied in combinations with other therapies such as well as studied for the treatment of solid tumors (226).

### 4.4 Current Strategies to Target Tumor-Associated Macrophages

TAMs can influence cancer relapse following treatment with conventional therapies; thus that several approaches have been developed to therapeutically target TAMs, from blocking the recruitment and infiltration of MDMs into the TME to interfering with TAM differentiation into tumor-promoting phenotypes and inhibiting proinflammatory cytokines and other stimuli responsible for chronic inflammation within the TME (5). Those therapies not only aim to block the ability of TAMs to promote cancer cell survival directly but can also increase cross-presentation to CD8+ T cells and thereby enhance their anti-tumoral potency.

Although TAMs are subject to tissue-specific imprinting, common strategies broadly target these cells across different organs, and many have shown promising results in preclinical models.

A considering number of these agents have entered clinical evaluation for diverse tumor types, including (i) inhibitors of CSF1R to deplete TAMs and/or alter their functions within the TME; (ii) CCL2 or CCR2 inhibitors to prevent TAMs recruitment into the TME; (iii) CD47/SIRPα complex antagonists to enhance TAM-mediated phagocytosis of cancer cells; (iv) administration of costimulatory molecules such as CD40 to enhance T-cell activation; (v) inhibitors of PI3Kγ and the triggering receptor expressed on myeloid cells 2 (TREM2) protein to reprogram TAMs toward anti-tumoral phenotypes; (vi) TLRs agonists to switch M2 phenotype into M1.

#### 4.4.1 CSF1 Inhibitors

CSF1R is a transmembrane tyrosine kinase class III receptor that has attracted interest primarily because it is exclusively expressed by cells of the monocytic lineage, and its specific ligand CSF1 (M-CSF) is required for macrophage differentiation and survival (227). Another known ligand is IL34, which role in cancer has been less explored partly due to its relatively recent identification as an alternative ligand (228). IL34 production by chemo-resistant lung cancer cells has been reported to enhance the immunosuppressive profile of TAMs and contribute to cancer cell survival (229). Also high levels of CSF1 circulating in the serum have been correlated with poor survival of patients, in particular those with ovarian and endometrial cancers (230) (**Figure 1B**).

Several drugs, from neutralizing antibodies to small-molecule inhibitors, directed against CSF1R have been used to deplete intratumoral TAMs or promote their re-education into a tumoricidal phenotype in a context-dependent manner (169, 231). In preclinical models of multiple primary tumors, including pancreatic cancer, breast cancer, and glioblastoma, this approach resulted in anti-tumor efficacy (186, 225) and reduced breast-to-lung metastasis (10).

For instance, the occurrence of melanoma brain metastasis was significantly hindered under microglia and macrophages elimination with PLX3397, a CSF-1R inhibitor; their depletion effectively inhibited the expression of matrix metalloproteinase 3 (MMP3) and the decrease of tight junction protein zonula occludens-1 (ZO-1), correlated with myeloid cells activation (171).

In contrast to previous findings from glioblastoma mouse models, where TAMs survived CSF1R inhibition and were instead re-educated (169, 170), a recent work demonstrated that targeting TAMs with the CSF1R inhibitor BLZ945 delayed brain metastatic onset and led to an initial tumor response with transient stasis of established metastases (232).CSF1R inhibitors have also been evaluated in combination treatments in preclinical studies. In breast cancer models, the efficacy of paclitaxel (Taxol) was enhanced by CSF1R inhibitor–mediated TAM depletion (10, 233). Similarly, the effectiveness of radiotherapy and tyrosine kinase inhibitors in preclinical glioblastoma models, when CSF1R is inhibited, seems to be mediated by TAMs re-education (13, 234). Preventing the entry of MDMs into the brain TME resulted in a comparatively modest effect in glioma models (13), indicating that TAMs reeducation more than their depletion may represent a more effective strategy (231).

Multiple drugs blocking CSF1R signaling (such as trastuzumab, ARRY-382, pexidartinib, PLX7486, and BLZ945) have been tested, including in combination with conventional therapies targeting cancer cells. Combinatorial strategies have also been explored in glioblastoma models where TAM populations where targeted with CSF1R inhibitor together with radiotherapy, enhancing survival of preclinical models (13). Others have also showed that CSF1R treatment prevent the accumulation of CD11b+Ly6C- monocytes, which recruitments is usually enhanced by radiation, limiting the pro-tumorigenic TAMs generation that supports tumor progression (235).

Several other clinical studies have been published and have reported different outcomes depending on the tumor type.

#### 4.4.2 CCL2/CCR2 Inhibitors

Chemokines usually drive monocyte recruitment and macrophages accumulation within the tumor and the expansion of the tissue-resident macrophage pool (236). In particular, CCL2 release by cancer cells leads to the recruitment not only of tissue-resident macrophages but also of CCR2+ Ly6C hi monocytes from the bloodstream that extravasate into tumor sites and differentiate into TAMs (237). High levels of CCL2 in the serum and the TME have often been associated with poor prognosis no matter the type of cancer (238, 239). Using neutralizing antibodies against CCL2 hindered the accumulation of TAMs and potentiated the anti-tumor efficacy of CD8+ T cells in the TME as the Ly6Chi monocytes were sequestered in the BM; thereby, it was shown a reduction in tumor growth and metastasis (146, 239) (**Figure 1B**). Although concerns about the long-term monotherapy efficacy were raised when its suspension triggered monocytes’ recruitment to the TME inducing lung metastasis and decreasing animals’ survival (240). Carlumab/CNTO888 (a human recombinant mAb targeting CCL2) entered phase I and II trials for patients with solid tumors, including metastatic castrate-resistant prostate cancer (NCT00992186 and NCT01204996), but despite being well-tolerated, it failed to affect tumor growth significantly, and the drug was discontinued. On the other hand, several anti-CCR2 mAbs have been tested in phase I and II trials for patients with bone metastasis (NCT01015560) and with advanced pancreatic adenocarcinoma (NCT01413022). More evidence supports the need for a thoughtful rationale to bring in the clinic using such therapies as combinatorial instead of monotherapy. For instance, PF-04136309 together with chemotherapy (FOLFIRINOX) resulted in a tumor response in 49% of the patients and local tumor control in 96% (241). The lack of evident clinical efficacy and the unexpected side effects may be explained by the CCL2 boost induced by the body made to overcome the inhibition of the CCL2/CCR2 axis (242) through still-unidentified compensatory mechanisms. Moreover, angiogenesis and local proliferation of resident TAMs may also dampen the effect of CCL2/CCR2 immunotherapy (240). Thus, focusing on new targets that selectively dampen monocytes’ recruitment and differentiation into pro-tumoral macrophages would be crucial to offering secondary options for unresponsive patients.

#### 4.4.3 CD47 Antagonists

Among many pro-tumoral functions exerted by TMAs can activate the immune response and even phagocyte cancer cells (4), for instance, the CD47-SIRPα interactions (243). CD47 is a “don’t eat me” immune checkpoint signaling receptor, which is constitutively expressed by normal cells and overexpressed on cancer cells (244), while CD47 binds signal regulatory protein α, expressed by TAMs, DCs and neutrophils (4). When SIRPα binds CD47, a cascade is initiated, inhibiting the phagocytic capacity of macrophages. Thus, it is believed to be important to block CD47–SIRPα interactions removes this inhibitory checkpoint signal augmenting the macrophage-mediated clearance of cancer cells (245), inducing DCs endocytosis and activation with the consequent T-cell mediated tumor clearance (243, 246, 247) (**Figure 1B**). In several preclinical models, this axis represents a promising innate immune checkpoint (243). Antibodies against CD47 are currently in the frontline of development, taken that magrolimab reduced mouse pediatric brain tumors (248), and a few others, like Hu5F9-G4, CC-90002, and ZL-1201, have started to be evaluated in patients. Along with those, several ongoing phases I studies for solid tumors and hematologic and B-cell malignancies (NCT03558139, NCT03248479, and NCT04599634), and a phase II trial (NCT02953782) for the treatment of solid tumors and advanced colorectal cancer has recently been completed but not shared yet.

#### 4.4.4 CD40 Agonists

CD40, a TNF receptor superfamily member, is expressed on APCs and is critical for their activation and proliferation, as well as an important regulator of T cell-dependent anti-tumor immunity *via* the interaction with CD40 ligand (CD40L) mainly expressed by CD4+ T cells (249). The signaling of this axis leads to secondary and tertiary signals for proper T-cell priming, such as the upregulation of MHC molecules, costimulatory molecules (like CD80 and CD86), and the production of proinflammatory cytokines (250, 251). For these reasons, CD40 can turn upside down the immune suppression and drive anti-tumor as its blockade induce the secretion of IFNγ and a tumoricidal phenotype as demonstrated in preclinical models of pancreatic cancer and patients with cancer (252). When CD40 agonists were used in combination with CSF1R inhibition, TAMs resulted as reprogrammed, reinforcing an effective T cell response (253). Many are the monoclonal agonistic antibodies (rhuCD40L, CP-870,893, and RO7009789) that are being evaluated in several clinical trials, and few with opposite results (**Figure 1B**). While a phase I trial of CP-870,893 in patients with advanced cancer showed no clinical responses (254), an early trial of rhuCD40L in patients with advanced squamous cell cancer of the head and neck had a broad spectrum of efficacy, with some showing modest responses and only one with a long-term remission (254). More recently, the tolerability and efficacy of a CD40 antibody (APX005M) combined with chemotherapy (gemcitabine plus nab-paclitaxel), with or without nivolumab, was achieved for patients with metastatic pancreatic adenocarcinoma in a phase Ib study (255). Overall, the scattered successes in cancer therapy of different patients leave undiscovered the biological reasons that must be explored to exploit the CD40 agonist as monotherapy or in combination with other ICIs.

#### 4.4.5 PI3K

The PI3Kγ is a myeloid-specific isoform of the PI3K family, which signaling pathway is important for regulating cell growth, survival, metabolism, angiogenesis, as its family members have important effects on the immune system. PI3Kγ acts as a key immunosuppressive pathway in myeloid cells, and its pharmacological inhibition has been studied in preclinical tumor models. Interestingly, PI3Kγ is a key regulator of TAM-mediated immunosuppression (256), and its selective inhibition increases MHC-II and IL12 expression and decreases IL10 in TAMs; as well as helping to overcome resistance to ICI, reshaping the TME and promoting CD8+ T cell recruitment and tumor regression (256, 257). Phase I and II clinical studies are now evaluating the inhibitor eganelisib (IPI-549) in diverse cancers, either as a monotherapy or in combination with ICI (NCT03719326, NCT02637531, NCT03795610, and NCT03980041). In 2020, the FDA granted eganelisib combined with ICI and chemotherapy for first-line treatment of patients with inoperable locally advanced or metastatic triple-negative breast cancer (NCT03961698). There are hundreds of ongoing clinical trials using pan-PI3K inhibitors, but only a few early-phase studies have employed specific inhibitors of the myeloid γ isoform in cancer patients (**Figure 1B**).

#### 4.4.6 TREM

This receptor is a member of the Ig superfamily and a major signaling hub with several proteins and ligands (258). The deficiency of TREM resulted in the reeducation of tumoral TAMs to an anti-tumoral phenotype (259, 260). TREM2 seems to be expressed in TAMs in more than 200 human cancer cases, and high levels correlate with poor outcomes in colorectal and breast cancers (259). An Ab (PY314) has been designed to deplete TREM expressing TAMs, and it is currently evaluated as monotherapy or in combination with pembrolizumab (NCT04691375) in a phase I trial (**Figure 1B**).

#### 4.4.7 TLRs

Toll-like receptors (TLRs), which are widely expressed by innate immune, are involved primarily in activating inflammatory immune responses. The first FDA-approved TLR agonist, subsequently used in combination with anti-PD1 therapy for bladder cancer, is Bacillus Calmette-Guerin (BCG), which triggers TLR2 and TLR4 (261, 262). Up to now, many are the pieces of evidence from *in vitro* and *in vivo* preclinical studies showing the potential activity of synthetic compounds specific for the endosomal TLR3, TLR7, TLR7, TLR8, and TLR9. Those ligands induce the secretion of immunostimulatory cytokines, like the type I IFN pathway, mostly in plasmacytoid DCs (pDCs) and macrophages (263), leading to an increased production of cytokines and infiltration of CD8+ T cells. To date, only imiquimod (TLR7 specific agonist) has been approved by the FDA for the topical treatment of squamous and basal cell carcinomas; others, like poly I: C (TLR3 agonist), resiquimod and NKTR-262 (TLR7/8 agonists), and CMP-001 and tilsotolimod (TLR9 agonist), have been developed and evaluated in early-phase clinical trials, either as adjuvants for cancer vaccines to boost anti-tumor responses or in combination with other treatments (264) (**Figure 1B**). Up to now, the topical application of TLR agonists in cutaneous neoplasms or intratumoral injection into accessible lesions has been thought safe.

The analog poly-ICLC is one of the most investigated compounds in more than 100 clinical trials and a few phases of I/II trials it has been exploited in combination with ICIs therapeutics in advanced diseases or as an anti-tumor vaccine adjuvant (265). An analog of imiquimod, resiquimod (TLR7/8 agonist), was shown to induce a strong anti-tumor response (266); either its topical administration or the local injection of loaded- nanoparticles induced tumor shrinkage and protective memory (267, 268). Intratumoral injection of MEDI9197 (3M-052) (specific for TLR7/TLR8) induced macrophage repolarization and tumor regression in a mouse model of subcutaneous melanoma; a mechanism mediated by macrophages induced direct tumor cell killing *via* NO production and synergized with checkpoint inhibitors anti-CTLA4 and anti-PD-1 antibodies to inhibit tumor growth (269, 270). Another one, SD-101 binds TLR9, has also been investigated in combination with immunotherapy (271, 272). Other TLR7/8 agonists are currently in phase III trial for skin neoplasia, anal carcinoma, and cervical intraepithelial lesions (264). For instance, TLR8 agonist motolimod, in combination with cetuximab, was shown to induce partial responses in a few patients with recurrent or metastatic head and neck cancer (273). Since most clinical trials have shown TLR agonists safe and promising in the clinic, such as tumor shrinkage after their injections, they would be probably more successful when used in combination with checkpoint inhibitors to treat those cold and non-responsive tumors.

## 5 New Relevant Targets

In recent years, an increasing number of studies have highlighted the important implications of **metabolism** on the biology and functional activities of immune cells. Despite the recent advances in this field, the metabolism of neutrophils is not fully understood and need further investigations, while it is already established that changes in metabolism generally shift macrophage polarization towards a tumor-promoting phenotype (274). Accordingly, metabolic modulation has been tested as a potential strategy to reprogram TAMs towards an anti-tumor state. Many tumor-derived metabolites have been discovered, such as adenosine, glutamine, and lactate, and have been mainly studied and tested in preclinical models to assess their effects on tumors. One of the most important findings has been the crucial role of glutamine. Thus, blocking its metabolism in a mouse breast cancer model reduced tumor growth and metastases, enhancing macrophage activation and inhibiting MDSC generation (275).

In parallel, an inhibitor of the enzyme glutamine synthase, named glufosinate, has been studied. In highly metastatic mouse models of melanoma and breast and lung cancer, it reduced metastasis formation, angiogenesis, and immunosuppression reprogramming TAMs into anti-tumor effectors (276). Furthermore, lactate is highly produced in hypoxic tumors and promotes M2 macrophage polarization (277) *via* activation of the ERK/STAT3 signaling pathway or the sensor protein Gpr81/Gpr132 expressed by macrophages. Pharmacological inhibition of the ERK/STAT3 axis with selumetinib or static or the Gpr132 protein hampered lactate-induced M2 macrophage polarization and showed significant anti-tumor effects in preclinical studies (278, 279) (**Figure 1C**).

A new study recently elucidated the modulating effects of lactic acid produced by tumor cells upon the macrophages within the TME. Transcriptomic and metabolic analyses have revealed two TAMs phenotypes with different metabolic features: the pro-inflammatory major histocompatibility complex (MHC)-II hi TAMs with a hampered tricarboxylic acid (TCA) cycle and the reparative MHC-II lo TAMs with higher oxidative and glycolytic metabolism. The latter population uses lactate as an additional carbon source besides glucose, supporting oxidative metabolism. This excess of carbon is partly compensated by the reduced uptake of glutamine and enhanced TCA cycle-mediated respiration. Additionally, it profoundly affects their transcriptome increases L-arginine-catabolizing enzymes, thus enhancing the T cell suppressive capacity of these TAMs (280).

Another tumor metabolite, adenosine, influences TAMs functions and, nonetheless, stimulation of adenosine receptors hinders the differentiation of monocytes to macrophages, probably through cAMP accumulation (281). Deletion of the adenosine receptor A2A in myeloid cells has been shown to prevent tumor progression and metastasis in melanoma tumor models (282), as well as its inhibition, enhances CD8+ T cells response in head and neck squamous cell carcinoma (283). This new field of research is appealing as well as challenging to explore as a therapeutic intervention in cancer patients as metabolic pathways are shared by all cells. Although metabolic macrophages rewiring could positively affect the combination with other treatments, the effects upon other cells of the TME need to be investigated, as well their long-term efficacy.

Other compounds have been tested, such as the histone deacetylase (HDAC) inhibitors. The selective class IIa inhibitor TMP195 has been proven to be successful in the epigenetic modulation of TAMs. In the MMTV-PyMT mammary tumor model, treatment with TMP195 stimulated macrophage-mediated phagocytosis of tumor cells and TAM reprogramming into proinflammatory immunostimulatory effectors (284). Combined treatment with TMP195 and chemotherapy or anti-PD-1 therapy resulted in increased anti-tumor effects. Additionally, a combination of low-dose adjuvant epigenetic compounds reduced metastatic spread in a preclinical metastasis model (after removing the primary tumor) (285), mainly mediated by the inhibition of myeloid cell recruitment in premetastatic niches.

Moreover, up to now, the only cell therapy approved by the FDA for hematological malignancies is the chimeric antigen receptor (CAR) T cells, genetically engineered to recognize the CD19 antigen (286). Still, the issues of the application to solid tumors are many. Classically, macrophages are more resistant to transduction procedures than lymphocytes and not many attempts to are being successful. Only one study has lightened hope, transducing an anti-HER2 CAR into primary human macrophages (CAR-Ms) (using a replication-incompetent adenovirus). They demonstrated in nude mice that the expression of the transgene reduces the volume of HER+ human tumors (287).

## 6 Concluding Remarks

A detailed understanding of the roles of myeloid cells in tumors has revealed their importance within the TME (5). Tumor-associated myeloid cells accumulate rapidly in tumors, where they constitute the largest population of leukocytes in tumors and, sometimes, outnumber tumor cells (256, 288). With a deeper understanding of cancer immunology, diverse strategies for the modulation of TAMs are being explored for therapeutic applications, while for neutrophils a specific strategy to modulate their phenotype has yet not been discovered; however, it is possible to affect their recruitment to ultimately avoid the detrimental effects usually observed upon their hijacking.

We must additionally look at the patient as a whole, considering the tumor as part of a circuit where microbiome, metabolism, obesity, lifestyle, and aging can alter the TME and affect treatment responsiveness.

Taken the diversity of the heterogeneity of myeloid cells within both primary tumors and their metastasis, and given the diverse targeting strategies currently used and underway, it is logical to think that characterization at the single-cell level should be included in the daily clinical practice to characterize and stratify each patient.

This approach would allow clinicians to find prognostic indicators and choose the most effective therapy based on the TME composition, lowering the cost of patient’s management for the healthcare sector, in the long run. This would not only support the development of personalized medicine but also exclude immune-related toxicity profiles of specific treatments.

## Author Contributions

The authors contributed equally to this work, with CN being senior and correspondent. MR, writing and editinga. CN, conceptualization, original draft preparation, writing, draw figure, and editing. All authors have read and agreed to the published version of the manuscript.

## Conflict of Interest

The authors declare that the research was conducted in the absence of any commercial or financial relationships that could be construed as a potential conflict of interest.

## Publisher’s Note

All claims expressed in this article are solely those of the authors and do not necessarily represent those of their affiliated organizations, or those of the publisher, the editors and the reviewers. Any product that may be evaluated in this article, or claim that may be made by its manufacturer, is not guaranteed or endorsed by the publisher.

## References

[1] Dewhirst MW Secomb TW. Transport of Drugs From Blood Vessels to Tumour Tissue. Nat Rev Cancer (2017) 17(12):738–50. doi: 10.1038/nrc.2017.93 PMC6371795 29123246

[2] Nia HT Munn LL Jain RK. Physical Traits of Cancer. In: Science (2020). Available at: https://www.science.org/doi/abs/10.1126/science.aaz0868. 370(6516) doi: 10.1126/science.aaz0868 PMC8274378 33122355

[3] Joyce JA. Therapeutic Targeting of the Tumor Microenvironment. Cancer Cell (2005) 7(6):513–20. doi: 10.1016/j.ccr.2005.05.024 15950901

[4] Mantovani A Marchesi F Malesci A Laghi L Allavena P. Tumour-Associated Macrophages as Treatment Targets in Oncology. Nat Rev Clin Oncol (2017) 14(7):399–416. doi: 10.1038/nrclinonc.2016.217 28117416 PMC5480600

[5] Cassetta L Pollard JW. Targeting Macrophages: Therapeutic Approaches in Cancer. Nat Rev Drug Discov (2018) 17(12):887–904. doi: 10.1038/nrd.2018.169 30361552

[6] Nakamura K Smyth MJ. Myeloid Immunosuppression and Immune Checkpoints in the Tumor Microenvironment. Cell Mol Immunol (2020) 17(1):1–12. doi: 10.1038/s41423-019-0306-1 31611651 PMC6952382

[7] Klemm F Joyce JA. Microenvironmental Regulation of Therapeutic Response in Cancer. Trends Cell Biol (2015) 25(4):198–213. doi: 10.1016/j.tcb.2014.11.006 25540894 PMC5424264

[8] Sharma P Hu-Lieskovan S Wargo JA Ribas A. Primary, Adaptive, and Acquired Resistance to Cancer Immunotherapy. Cell (2017) 168(4):707–23. doi: 10.1016/j.cell.2017.01.017 PMC5391692 28187290

[9] Weissleder R Pittet MJ. The Expanding Landscape of Inflammatory Cells Affecting Cancer Therapy. Nat BioMed Eng (2020) 4(5):489–98. doi: 10.1038/s41551-020-0524-y PMC7405944 32203281

[10] DeNardo DG Brennan DJ Rexhepaj E Ruffell B Shiao SL Madden SF. Leukocyte Complexity Predicts Breast Cancer Survival and Functionally Regulates Response to Chemotherapy. Cancer Discov (2011) 1(1):54–67. doi: 10.1158/2159-8274.CD-10-0028 22039576 PMC3203524

[11] Takeuchi S Baghdadi M Tsuchikawa T Wada H Nakamura T Abe H. Chemotherapy-Derived Inflammatory Responses Accelerate the Formation of Immunosuppressive Myeloid Cells in the Tissue Microenvironment of Human Pancreatic Cancer. Cancer Res (2015) 75(13):2629–40. doi: 10.1158/0008-5472.CAN-14-2921 25952647

[12] Seifert L Werba G Tiwari S Giao Ly NN Nguy S Alothman S. Radiation Therapy Induces Macrophages to Suppress T-Cell Responses Against Pancreatic Tumors in Mice. Gastroenterology (2016) 150(7):1659–72. doi: 10.1053/j.gastro.2016.02.070 PMC4909514 26946344

[13] Akkari L Bowman RL Tessier J Klemm F Handgraaf SM de Groot M. Dynamic Changes in Glioma Macrophage Populations After Radiotherapy Reveal CSF-1R Inhibition as a Strategy to Overcome Resistance. Sci Trans Med (2020) 12(552):eaaw7843. doi: 10.1126/scitranslmed.aaw7843 32669424

[14] Sun Y Campisi J Higano C Beer TM Porter P Coleman I. Treatment-Induced Damage to the Tumor Microenvironment Promotes Prostate Cancer Therapy Resistance Through WNT16B. Nat Med (2012) 18(9):1359–68. doi: 10.1038/nm.2890 PMC3677971 22863786

[15] Moeller BJ Cao Y Li CY Dewhirst MW. Radiation Activates HIF-1 to Regulate Vascular Radiosensitivity in Tumors: Role of Reoxygenation, Free Radicals, and Stress Granules. Cancer Cell (2004) 5(5):429–41. doi: 10.1016/S1535-6108(04)00115-1 15144951

[16] Vincent J Mignot G Chalmin F Ladoire S Bruchard M Chevriaux A. 5-Fluorouracil Selectively Kills Tumor-Associated Myeloid-Derived Suppressor Cells Resulting in Enhanced T Cell-Dependent Antitumor Immunity. Cancer Res (2010) 70(8):3052–61. doi: 10.1158/0008-5472.CAN-09-3690 20388795

[17] Galluzzi L Humeau J Buqué A Zitvogel L Kroemer G. Immunostimulation With Chemotherapy in the Era of Immune Checkpoint Inhibitors. Nat Rev Clin Oncol (2020) 17(12):725–41. doi: 10.1038/s41571-020-0413-z 32760014

[18] Balkwill F Mantovani A. Inflammation and Cancer: Back to Virchow? Lancet (2001) 357(9255):539–45. doi: 10.1016/S0140-6736(00)04046-0 11229684

[19] Robert J. Comparative Study of Tumorigenesis and Tumor Immunity in Invertebrates and Nonmammalian Vertebrates. Dev Comp Immunol (2010) 34(9):915–25. doi: 10.1016/j.dci.2010.05.011 PMC2900388 20553753

[20] Wang J Cao Z Zhang X-M Nakamura M Sun M Hartman J. Novel Mechanism of Macrophage-Mediated Metastasis Revealed in a Zebrafish Model of Tumor Development. Cancer Res (2015) 75(2):306–15. doi: 10.1158/0008-5472.CAN-14-2819 25492861

[21] Topalian SL Drake CG Pardoll DM. Immune Checkpoint Blockade: A Common Denominator Approach to Cancer Therapy. Cancer Cell (2015) 27(4):450–61. doi: 10.1016/j.ccell.2015.03.001 PMC4400238 25858804

[22] Ruffell B Coussens LM. Macrophages and Therapeutic Resistance in Cancer. Cancer Cell (2015) 27(4):462–72. doi: 10.1016/j.ccell.2015.02.015 PMC4400235 25858805

[23] Allavena P Digifico E Belgiovine C. Macrophages and Cancer Stem Cells: A Malevolent Alliance. Mol Med (2021) 27(1):121. doi: 10.1186/s10020-021-00383-3 34583655 PMC8480058

[24] Noy R Pollard JW. Tumor-Associated Macrophages: From Mechanisms to Therapy. Immunity (2014) 41(1):49–61. doi: 10.1016/j.immuni.2014.06.010 25035953 PMC4137410

[25] Mantovani A Marchesi F Jaillon S Garlanda C Allavena P. Tumor-Associated Myeloid Cells: Diversity and Therapeutic Targeting. Cell Mol Immunol (2021) 18(3):566–78. doi: 10.1038/s41423-020-00613-4 PMC8027665 33473192

[26] Ng LG Ostuni R Hidalgo A. Heterogeneity of Neutrophils. Nat Rev Immunol (2019) 19(4):255–65. doi: 10.1038/s41577-019-0141-8 30816340

[27] O’Connell KE Mikkola AM Stepanek AM Vernet A Hall CD Sun CC. Practical Murine Hematopathology: A Comparative Review and Implications for Research. Comp Med (2015) 65(2):96–113. 25926395 PMC4408895

[28] Martin KR Wong HL Witko-Sarsat V Wicks IP. G-CSF – A Double Edge Sword in Neutrophil Mediated Immunity. Semin Immunol (2021) 54:101516. doi: 10.1016/j.smim.2021.101516 34728120

[29] Eash KJ Greenbaum AM Gopalan PK Link DC. CXCR2 and CXCR4 Antagonistically Regulate Neutrophil Trafficking From Murine Bone Marrow. J Clin Invest (2010) 120(7):2423–31. doi: 10.1172/JCI41649 PMC2898597 20516641

[30] Manz MG Boettcher S. Emergency Granulopoiesis. Nat Rev Immunol (2014) 14(5):302–14. doi: 10.1038/nri3660 24751955

[31] Evrard M Kwok IWH Chong SZ Teng KWW Becht E Chen J. Developmental Analysis of Bone Marrow Neutrophils Reveals Populations Specialized in Expansion, Trafficking, and Effector Functions. Immunity (2018) 48(2):364–379.e8. doi: 10.1016/j.immuni.2018.02.002 29466759

[32] Mumau MD Vanderbeck AN Lynch ED Golec SB Emerson SG Punt JA. Identification of a Multipotent Progenitor Population in the Spleen That Is Regulated by NR4A1. J Immunol (2018) 200(3):1078–87. doi: 10.4049/jimmunol.1701250 PMC5793861 29282309

[33] Xie X Shi Q Wu P Zhang X Kambara H Su J. Single-Cell Transcriptome Profiling Reveals Neutrophil Heterogeneity in Homeostasis and Infection. Nat Immunol (2020) 21(9):1119–33. doi: 10.1038/s41590-020-0736-z PMC7442692 32719519

[34] Baggiolini M Walz A Kunkel SL. Neutrophil-Activating Peptide-1/Interleukin 8, a Novel Cytokine That Activates Neutrophils. J Clin Invest (1989) 84(4):1045–9. doi: 10.1172/JCI114265 PMC329758 2677047

[35] Zhu YP Padgett L Dinh HQ Marcovecchio P Blatchley A Wu R. Identification of an Early Unipotent Neutrophil Progenitor With Pro-Tumoral Activity in Mouse and Human Bone Marrow. Cell Rep (2018) 24(9):2329–41.e8. doi: 10.1016/j.celrep.2018.07.097 30157427 PMC6542273

[36] Ley K Hoffman HM Kubes P Cassatella MA Zychlinsky A Hedrick CC. Neutrophils: New Insights and Open Questions. Sci Immunol (2018) 3(30). doi: 10.1126/sciimmunol.aat4579 30530726

[37] Abi Abdallah DS Egan CE Butcher BA Denkers EY. Mouse Neutrophils Are Professional Antigen-Presenting Cells Programmed to Instruct Th1 and Th17 T-Cell Differentiation. Int Immunol (2011) 23(5):317–26. doi: 10.1093/intimm/dxr007 PMC3082529 21422151

[38] Vono M Lin A Norrby-Teglund A Koup RA Liang F Loré K. Neutrophils Acquire the Capacity for Antigen Presentation to Memory CD4+ T Cells *In Vitro* and *Ex Vivo* . Blood (2017) 129(14):1991–2001. doi: 10.1182/blood-2016-10-744441 28143882 PMC5383872

[39] Nicolás-Ávila JÁ Adrover JM Hidalgo A. Neutrophils in Homeostasis, Immunity, and Cancer. Immunity (2017) 46(1):15–28. doi: 10.1016/j.immuni.2016.12.012 28099862

[40] Jaillon S Ponzetta A Di Mitri D Santoni A Bonecchi R Mantovani A. Neutrophil Diversity and Plasticity in Tumour Progression and Therapy. Nat Rev Cancer (2020) 20(9):485–503. doi: 10.1038/s41568-020-0281-y 32694624

[41] Finisguerra V Di Conza G Di Matteo M Serneels J Costa S Thompson AAR. MET Is Required for the Recruitment of Anti-Tumoural Neutrophils. Nature (2015) 522(7556):349–53. doi: 10.1038/nature14407 PMC4594765 25985180

[42] Cui C Chakraborty K Tang XA Zhou G Schoenfelt KQ Becker KM. Neutrophil Elastase Selectively Kills Cancer Cells and Attenuates Tumorigenesis. Cell (2021) 184(12):3163–3177.e21. doi: 10.1016/j.cell.2021.04.016 33964209 PMC10712736

[43] Blaisdell A Crequer A Columbus D Daikoku T Mittal K Dey SK. Neutrophils Oppose Uterine Epithelial Carcinogenesis *via* Debridement of Hypoxic Tumor Cells. Cancer Cell (2015) 28(6):785–99. doi: 10.1016/j.ccell.2015.11.005 PMC4698345 26678340

[44] Jamieson T Clarke M Steele CW Samuel MS Neumann J Jung A. Inhibition of CXCR2 Profoundly Suppresses Inflammation-Driven and Spontaneous Tumorigenesis. J Clin Invest (2012) 122(9):3127–44. doi: 10.1172/JCI61067 PMC3428079 22922255

[45] Khaw YM Cunningham C Tierney A Sivaguru M Inoue M. Neutrophil-Selective Deletion of Cxcr2 Protects Against CNS Neurodegeneration in a Mouse Model of Multiple Sclerosis. J Neuroinflammation (2020) 17(1):49. doi: 10.1186/s12974-020-1730-y 32019585 PMC7001284

[46] Ponzetta A Carriero R Carnevale S Barbagallo M Molgora M Perucchini C. Neutrophils Driving Unconventional T Cells Mediate Resistance Against Murine Sarcomas and Selected Human Tumors. Cell (2019) 178(2):346–360.e24. doi: 10.1016/j.cell.2019.05.047 31257026 PMC6630709

[47] Singhal S Bhojnagarwala PS O’Brien S Moon EK Garfall AL Rao AS. Origin and Role of a Subset of Tumor-Associated Neutrophils With Antigen-Presenting Cell Features in Early-Stage Human Lung Cancer. Cancer Cell (2016) 30(1):120–35. doi: 10.1016/j.ccell.2016.06.001 PMC4945447 27374224

[48] Granot Z Henke E Comen EA King TA Norton L Benezra R. Tumor Entrained Neutrophils Inhibit Seeding in the Premetastatic Lung. Cancer Cell (2011) 20(3):300–14. doi: 10.1016/j.ccr.2011.08.012 PMC3172582 21907922

[49] López-Lago MA Posner S Thodima VJ Molina AM Motzer RJ Chaganti RSK. Neutrophil Chemokines Secreted by Tumor Cells Mount a Lung Antimetastatic Response During Renal Cell Carcinoma Progression. Oncogene (2013) 32(14):1752–60. doi: 10.1038/onc.2012.201 PMC3435490 22665059

[50] Massara M Bonavita O Savino B Caronni N Poeta VM Sironi M. ACKR2 in Hematopoietic Precursors as a Checkpoint of Neutrophil Release and Anti-Metastatic Activity. Nat Commun (2018) 9(1):676. doi: 10.1038/s41467-018-03080-8 29445158 PMC5813042

[51] Balkwill FR Mantovani A. Cancer-Related Inflammation: Common Themes and Therapeutic Opportunities. Semin Cancer Biol (2012) 22(1):33–40. doi: 10.1016/j.semcancer.2011.12.005 22210179

[52] Nastasi C Mannarino L D’Incalci M. DNA Damage Response and Immune Defense. Int J Mol Sci (2020) 21(20):7504. doi: 10.3390/ijms21207504 PMC7588887 33053746

[53] De Palma M Biziato D Petrova TV. Microenvironmental Regulation of Tumour Angiogenesis. Nat Rev Cancer (2017) 17(8):457–74. doi: 10.1038/nrc.2017.51 28706266

[54] Wculek SK Bridgeman VL Peakman F Malanchi I. Early Neutrophil Responses to Chemical Carcinogenesis Shape Long-Term Lung Cancer Susceptibility. iScience (2020) 23(7):101277. doi: 10.1016/j.isci.2020.101277 32619702 PMC7334367

[55] Canli Ö Nicolas AM Gupta J Finkelmeier F Goncharova O Pesic M. Myeloid Cell-Derived Reactive Oxygen Species Induce Epithelial Mutagenesis. Cancer Cell (2017) 32(6):869–883.e5. doi: 10.1016/j.ccell.2017.11.004 29232557

[56] Russo M Giavazzi R. Anti-Angiogenesis for Cancer: Current Status and Prospects. Thromb Res (2018) 164:Supplement 1:S3–6. doi: 10.1016/j.thromres.2018.01.030 29703482

[57] Albini A Bruno A Noonan DM Mortara L. Contribution to Tumor Angiogenesis From Innate Immune Cells Within the Tumor Microenvironment: Implications for Immunotherapy. Front Immunol (2018) 9:527. doi: 10.3389/fimmu.2018.00527 29675018 PMC5895776

[58] Haibe Y Kreidieh M El Hajj H Khalifeh I Mukherji D Temraz S. Resistance Mechanisms to Anti-Angiogenic Therapies in Cancer. Front Oncol (2020) 10:221. doi: 10.3389/fonc.2020.00221 32175278 PMC7056882

[59] Shojaei F Wu X Zhong C Yu L Liang X-H Yao J. Bv8 Regulates Myeloid-Cell-Dependent Tumour Angiogenesis. Nature (2007) 450(7171):825–31. doi: 10.1038/nature06348 18064003

[60] Belotti D Pinessi D Taraboletti G. Alternative Vascularization Mechanisms in Tumor Resistance to Therapy. Cancers (2021) 13(8):1912. doi: 10.3390/cancers13081912 33921099 PMC8071410

[61] Spiegel A Brooks MW Houshyar S Reinhardt F Ardolino M Fessler E. Neutrophils Suppress Intraluminal NK Cell-Mediated Tumor Cell Clearance and Enhance Extravasation of Disseminated Carcinoma Cells. Cancer Discov (2016) 6(6):630–49. doi: 10.1158/2159-8290.CD-15-1157 PMC4918202 27072748

[62] Li P Lu M Shi J Hua L Gong Z Li Q. Dual Roles of Neutrophils in Metastatic Colonization are Governed by the Host NK Cell Status. Nat Commun (2020) 11(1):4387. doi: 10.1038/s41467-020-18125-0 32873795 PMC7463263

[63] Wculek SK Malanchi I. Neutrophils Support Lung Colonization of Metastasis-Initiating Breast Cancer Cells. Nature (2015) 528(7582):413–7. doi: 10.1038/nature16140 PMC4700594 26649828

[64] Coffelt SB Kersten K Doornebal CW Weiden J Vrijland K Hau C-S. IL-17-Producing γδ T Cells and Neutrophils Conspire to Promote Breast Cancer Metastasis. Nature (2015) 522(7556):345–8. doi: 10.1038/nature14282 PMC4475637 25822788

[65] Szczerba BM Castro-Giner F Vetter M Krol I Gkountela S Landin J. Neutrophils Escort Circulating Tumour Cells to Enable Cell Cycle Progression. Nature (2019) 566:553–57. doi: 10.1038/s41586-019-0915-y 30728496

[66] Liang W Li Q Ferrara N. Metastatic Growth Instructed by Neutrophil-Derived Transferrin. PNAS (2018) 115(43):11060–5. doi: 10.1073/pnas.1811717115 PMC6205468 30301793

[67] Faget J Groeneveld S Boivin G Sankar M Zangger N Garcia M. Neutrophils and Snail Orchestrate the Establishment of a Pro-Tumor Microenvironment in Lung Cancer. Cell Rep (2017) 21(11):3190–204. doi: 10.1016/j.celrep.2017.11.052 29241546

[68] Jackstadt R van Hooff SR Leach JD Cortes-Lavaud X Lohuis JO Ridgway RA. Epithelial NOTCH Signaling Rewires the Tumor Microenvironment of Colorectal Cancer to Drive Poor-Prognosis Subtypes and Metastasis. Cancer Cell (2019) 36(3):319–36.e7. doi: 10.1016/j.ccell.2019.08.003 31526760 PMC6853173

[69] Markman JL Porritt RA Wakita D Lane ME Martinon D Noval Rivas M. Loss of Testosterone Impairs Anti-Tumor Neutrophil Function. Nat Commun (2020) 11(1):1613. doi: 10.1038/s41467-020-15397-4 32235862 PMC7109066

[70] Papayannopoulos V. Neutrophil Extracellular Traps in Immunity and Disease. Nat Rev Immunol (2018) 18(2):134–47. doi: 10.1038/nri.2017.105 28990587

[71] Albrengues J Shields MA Ng D Park CG Ambrico A Poindexter ME. Neutrophil Extracellular Traps Produced During Inflammation Awaken Dormant Cancer Cells in Mice. Science (2018) 361(6409):eaao4227. doi: 10.1126/science.aao4227 30262472 PMC6777850

[72] Park J Wysocki RW Amoozgar Z Maiorino L Fein MR Jorns J. Cancer Cells Induce Metastasis-Supporting Neutrophil Extracellular DNA Traps. Sci Transl Med (2016) 8(361):361ra138. doi: 10.1126/scitranslmed.aag1711 PMC5550900 27798263

[73] Lee W Ko SY Mohamed MS Kenny HA Lengyel E Naora H. Neutrophils Facilitate Ovarian Cancer Premetastatic Niche Formation in the Omentum. J Exp Med (2019) 216(1):176–94. doi: 10.1084/jem.20181170 PMC6314534 30567719

[74] Yang L-Y Luo Q Lu L Zhu W-W Sun H-T Wei R. Increased Neutrophil Extracellular Traps Promote Metastasis Potential of Hepatocellular Carcinoma *via* Provoking Tumorous Inflammatory Response. J Hematol Oncol (2020) 13(1):3. doi: 10.1186/s13045-019-0836-0 31907001 PMC6945602

[75] He X Xu C. Immune Checkpoint Signaling and Cancer Immunotherapy. Cell Res (2020) 30(8):660–9. doi: 10.1038/s41422-020-0343-4 PMC7395714 32467592

[76] Kroemer G Zitvogel L. Immune Checkpoint Inhibitors. J Exp Med (2021) 218(3):e20201979. doi: 10.1084/jem.20201979 33600556 PMC7888351

[77] Jenkins RW Barbie DA Flaherty KT. Mechanisms of Resistance to Immune Checkpoint Inhibitors. Br J Cancer (2018) 118(1):9–16. doi: 10.1038/bjc.2017.434 29319049 PMC5765236

[78] Bruni D Angell HK Galon J. The Immune Contexture and Immunoscore in Cancer Prognosis and Therapeutic Efficacy. Nat Rev Cancer (2020) 20(11):662–80. doi: 10.1038/s41568-020-0285-7 32753728

[79] Faget J Peters S Quantin X Meylan E Bonnefoy N. Neutrophils in the Era of Immune Checkpoint Blockade. J Immunother Cancer (2021) 9(7):e002242. doi: 10.1136/jitc-2020-002242 34301813 PMC8728357

[80] Bullock K Richmond A. Suppressing MDSC Recruitment to the Tumor Microenvironment by Antagonizing CXCR2 to Enhance the Efficacy of Immunotherapy. Cancers (2021) 13(24):6293. doi: 10.3390/cancers13246293 34944914 PMC8699249

[81] Highfill SL Cui Y Giles AJ Smith JP Zhang H Morse E. Disruption of CXCR2-Mediated MDSC Tumor Trafficking Enhances Anti-PD1 Efficacy. Sci Transl Med (2014) 6(237):237ra67. doi: 10.1126/scitranslmed.3007974 PMC6980372 24848257

[82] Sun L Clavijo PE Robbins Y Patel P Friedman J Greene S. Inhibiting Myeloid-Derived Suppressor Cell Trafficking Enhances T Cell Immunotherapy. JCI Insight (2019) 4(7):e126853. doi: 10.1172/jci.insight.126853 PMC6483637 30944253

[83] Syntrix Biosystems, Inc. A Phase 1, Open-Label, Dose-Escalation With Expansion Study of SX-682 in Subjects With Metastatic Melanoma Concurrently Treated With Pembrolizumab. clinicaltrials.gov (2021). Available at: https://clinicaltrials.gov/ct2/show/NCT03161431. Report No.: NCT03161431.

[84] Dunne R. An Open-Label Phase 1 Study to Evaluate the Safety and Tolerability of SX-682 in Combination With Nivolumab as a Maintenance Therapy in Patients With Metastatic Pancreatic Ductal Adenocarcinoma. clinicaltrials.gov (2021). Available at: https://clinicaltrials.gov/ct2/show/NCT04477343. Report No.: NCT04477343.

[85] M.D. Anderson Cancer Center. Phase Ib/II Trial of SX-682 in Combination With Nivolumab for Refractory RAS Mutated (RAS) Microsatellite Stable (MSS) Metastatic Colorectal Cancer (mCRC) (STOPTRAFFIC-1). clinicaltrials.gov (2021). Available at: https://clinicaltrials.gov/ct2/show/NCT04599140. Report No.: NCT04599140.

[86] Biasci D Smoragiewicz M Connell CM Wang Z Gao Y Thaventhiran JED. CXCR4 Inhibition in Human Pancreatic and Colorectal Cancers Induces an Integrated Immune Response. PNAS (2020) 117(46):28960–70. doi: 10.1073/pnas.2013644117 PMC7682333 33127761

[87] M.D. Anderson Cancer Center. A Phase IIb Pilot Study to Assess the Efficacy, Safety, and Pharmacodynamics Effects of Pembrolizumab and BL-8040 in Patients With Metastatic Pancreatic Cancer. clinicaltrials.gov (2020). Available at: https://clinicaltrials.gov/ct2/show/NCT02907099. Report No.: NCT02907099.

[88] Liu Q Li Z Gao J-L Wan W Ganesan S McDermott DH. CXCR4 Antagonist AMD3100 Redistributes Leukocytes From Primary Immune Organs to Secondary Immune Organs, Lung, and Blood in Mice. Eur J Immunol (2015) 45(6):1855–67. doi: 10.1002/eji.201445245 PMC4461468 25801950

[89] Devi S Wang Y Chew WK Lima R A-González N Mattar CNZ. Neutrophil Mobilization *via* Plerixafor-Mediated CXCR4 Inhibition Arises From Lung Demargination and Blockade of Neutrophil Homing to the Bone Marrow. J Exp Med (2013) 210(11):2321–36. doi: 10.1084/jem.20130056 PMC3804935 24081949

[90] Fridlender ZG Sun J Kim S Kapoor V Cheng G Ling L. Polarization of Tumor-Associated Neutrophil Phenotype by TGF-Beta: “N1” Versus “N2” TAN. Cancer Cell (2009) 16(3):183–94. doi: 10.1016/j.ccr.2009.06.017 PMC2754404 19732719

[91] Horn LA Riskin J Hempel HA Fousek K Lind H Hamilton DH. Simultaneous Inhibition of CXCR1/2, TGF-β, and PD-L1 Remodels the Tumor and Its Microenvironment to Drive Antitumor Immunity. J Immunother Cancer (2020) 8(1):e000326. doi: 10.1136/jitc-2019-000326 32188703 PMC7078948

[92] National Cancer Institute (NCI). Phase I/II Trial Investigating the Safety, Tolerability, Pharmacokinetics, Immune and Clinical Activity of SX-682 in Combination With BinTrafusp Alfa (M7824 or TGF-Beta “Trap”/PD-L1) With CV301 TRICOM in Advanced Solid Tumors (STAT). clinicaltrials.gov (2021). Available at: https://clinicaltrials.gov/ct2/show/NCT04574583. Report No.: NCT04574583.

[93] Zhang M Huang L Ding G Huang H Cao G Sun X. Interferon Gamma Inhibits CXCL8-CXCR2 Axis Mediated Tumor-Associated Macrophages Tumor Trafficking and Enhances Anti-PD1 Efficacy in Pancreatic Cancer. J Immunother Cancer (2020) 8(1):e000308. doi: 10.1136/jitc-2019-000308 32051287 PMC7057481

[94] Fox Chase Cancer Center. Combination Immunotherapy With Interferon-Gamma and Nivolumab for Patients With Advanced Solid Tumors: A Phase 1 Study. clinicaltrials.gov (2019). Available at: https://clinicaltrials.gov/ct2/show/NCT02614456. Report No.: NCT02614456.

[95] Shojaei F Wu X Malik AK Zhong C Baldwin ME Schanz S. Tumor Refractoriness to Anti-VEGF Treatment Is Mediated by CD11b+Gr1+ Myeloid Cells. Nat Biotechnol (2007) 25(8):911–20. doi: 10.1038/nbt1323 17664940

[96] Itatani Y Yamamoto T Zhong C Molinolo AA Ruppel J Hegde P. Suppressing Neutrophil-Dependent Angiogenesis Abrogates Resistance to Anti-VEGF Antibody in a Genetic Model of Colorectal Cancer. PNAS (2020) 117(35):21598–608. doi: 10.1073/pnas.2008112117 PMC7474657 32817421

[97] Schiffmann LM Fritsch M Gebauer F Günther SD Stair NR Seeger JM. Tumour-Infiltrating Neutrophils Counteract Anti-VEGF Therapy in Metastatic Colorectal Cancer. Br J Cancer (2019) 120(1):69–78. doi: 10.1038/s41416-018-0198-3 30377339 PMC6325148

[98] Liang J Piao Y Holmes L Fuller GN Henry V Tiao N. Neutrophils Promote the Malignant Glioma Phenotype Through S100A4. Clin Cancer Res (2014) 20(1):187–98. doi: 10.1158/1078-0432.CCR-13-1279 PMC4422653 24240114

[99] Galdiero MR Bianchi P Grizzi F Di Caro G Basso G Ponzetta A. Occurrence and Significance of Tumor-Associated Neutrophils in Patients With Colorectal Cancer. Int J Cancer (2016) 139(2):446–56. doi: 10.1002/ijc.30076 26939802

[100] Zhou G Peng K Song Y Yang W Shu W Yu T. CD177+ Neutrophils Suppress Epithelial Cell Tumourigenesis in Colitis-Associated Cancer and Predict Good Prognosis in Colorectal Cancer. Carcinogenesis (2018) 39(2):272–82. doi: 10.1093/carcin/bgx142 29228136

[101] Shaul ME Fridlender ZG. Tumour-Associated Neutrophils in Patients With Cancer. Nat Rev Clin Oncol (2019) 16(10):601–20. doi: 10.1038/s41571-019-0222-4 31160735

[102] Zhou S-L Zhou Z-J Hu Z-Q Huang X-W Wang Z Chen E-B. Tumor-Associated Neutrophils Recruit Macrophages and T-Regulatory Cells to Promote Progression of Hepatocellular Carcinoma and Resistance to Sorafenib. Gastroenterology (2016) 150(7):1646–1658.e17. doi: 10.1053/j.gastro.2016.02.040 26924089

[103] Arvanitakis K Mitroulis I Germanidis G. Tumor-Associated Neutrophils in Hepatocellular Carcinoma Pathogenesis, Prognosis, and Therapy. Cancers (2021) 13(12):2899. doi: 10.3390/cancers13122899 34200529 PMC8228651

[104] Cheng Y Mo F Li Q Han X Shi H Chen S. Targeting CXCR2 Inhibits the Progression of Lung Cancer and Promotes Therapeutic Effect of Cisplatin. Mol Cancer (2021) 20(1):62. doi: 10.1186/s12943-021-01355-1 33814009 PMC8019513

[105] Nywening TM Belt BA Cullinan DR Panni RZ Han BJ Sanford DE. Targeting Both Tumour-Associated CXCR2+ Neutrophils and CCR2+ Macrophages Disrupts Myeloid Recruitment and Improves Chemotherapeutic Responses in Pancreatic Ductal Adenocarcinoma. Gut (2018) 67(6):1112–23. doi: 10.1136/gutjnl-2017-313738 PMC5969359 29196437

[106] Bhattacharya U Gutter-Kapon L Kan T Boyango I Barash U Yang S-M. Heparanase and Chemotherapy Synergize to Drive Macrophage Activation and Enhance Tumor Growth. Cancer Res (2020) 80(1):57–68. doi: 10.1158/0008-5472.CAN-19-1676 31690669 PMC6942624

[107] Crawford J Dale DC Lyman GH. Chemotherapy-Induced Neutropenia. Cancer (2004) 100(2):228–37. doi: 10.1002/cncr.11882 14716755

[108] Lalami Y Klastersky J. Impact of Chemotherapy-Induced Neutropenia (CIN) and Febrile Neutropenia (FN) on Cancer Treatment Outcomes: An Overview About Well-Established and Recently Emerging Clinical Data. Crit Rev Oncol Hematol (2017) 120:163–79. doi: 10.1016/j.critrevonc.2017.11.005 29198330

[109] Feins S Kong W Williams EF Milone MC Fraietta JA. An Introduction to Chimeric Antigen Receptor (CAR) T-Cell Immunotherapy for Human Cancer. Am J Hematol (2019) 94(S1):S3–9. doi: 10.1002/ajh.25418 30680780

[110] Veglia F Tyurin VA Blasi M De Leo A Kossenkov AV Donthireddy L. Fatty Acid Transport Protein 2 Reprograms Neutrophils in Cancer. Nature (2019) 569(7754):73–8. doi: 10.1038/s41586-019-1118-2 PMC6557120 30996346

[111] Matlung HL Babes L Zhao XW van Houdt M Treffers LW van Rees DJ. Neutrophils Kill Antibody-Opsonized Cancer Cells by Trogoptosis. Cell Rep (2018) 23(13):3946–3959.e6. doi: 10.1016/j.celrep.2018.05.082 29949776

[112] Wynn TA Chawla A Pollard JW. Macrophage Biology in Development, Homeostasis and Disease. Nature (2013) 496(7446):445–55. doi: 10.1038/nature12034 PMC3725458 23619691

[113] Yona S Kim K-W Wolf Y Mildner A Varol D Breker M. Fate Mapping Reveals Origins and Dynamics of Monocytes and Tissue Macrophages Under Homeostasis. Immunity (2013) 38(1):79–91. doi: 10.1016/j.immuni.2012.12.001 23273845 PMC3908543

[114] Gordon S Plüddemann A. The Mononuclear Phagocytic System. Gener Divers Front Immunol (2019) 10:1893. doi: 10.3389/fimmu.2019.01893 PMC6696592 31447860

[115] Murray PJ Allen JE Biswas SK Fisher EA Gilroy DW Goerdt S. Macrophage Activation and Polarization: Nomenclature and Experimental Guidelines. Immunity (2014) 41(1):14–20. doi: 10.1016/j.immuni.2014.06.008 25035950 PMC4123412

[116] Davies LC Jenkins SJ Allen JE Taylor PR. Tissue-Resident Macrophages. Nat Immunol (2013) 14(10):986–95. doi: 10.1038/ni.2705 PMC4045180 24048120

[117] Schulz C Gomez Perdiguero E Chorro L Szabo-Rogers H Cagnard N Kierdorf K. A Lineage of Myeloid Cells Independent of Myb and Hematopoietic Stem Cells. Science (2012) 336(6077):86–90. doi: 10.1126/science.1219179 22442384

[118] Hoeffel G Chen J Lavin Y Low D Almeida FF See P. C-Myb(+) Erythro-Myeloid Progenitor-Derived Fetal Monocytes Give Rise to Adult Tissue-Resident Macrophages. Immunity (2015) 42(4):665–78. doi: 10.1016/j.immuni.2015.03.011 PMC4545768 25902481

[119] Bowman RL Klemm F Akkari L Pyonteck SM Sevenich L Quail DF. Macrophage Ontogeny Underlies Differences in Tumor-Specific Education in Brain Malignancies. Cell Rep (2016) 17(9):2445–59. doi: 10.1016/j.celrep.2016.10.052 PMC5450644 27840052

[120] Ajami B Bennett JL Krieger C Tetzlaff W Rossi FMV. Local Self-Renewal can Sustain CNS Microglia Maintenance and Function Throughout Adult Life. Nat Neurosci (2007) 10(12):1538–43. doi: 10.1038/nn2014 18026097

[121] Goldmann J Kwidzinski E Brandt C Mahlo J Richter D Bechmann I. T Cells Traffic From Brain to Cervical Lymph Nodes *via* the Cribroid Plate and the Nasal Mucosa. J Leuk Biol (2006) 80(4):797–801. doi: 10.1189/jlb.0306176 16885505

[122] Shi C Pamer EG. Monocyte Recruitment During Infection and Inflammation. Nat Rev Immunol (2011) 11(11):762–74. doi: 10.1038/nri3070 PMC3947780 21984070

[123] Mass E Ballesteros I Farlik M Halbritter F Günther P Crozet L. Specification of Tissue-Resident Macrophages During Organogenesis. Science (2016) 353(6304):aaf4238. doi: 10.1126/science.aaf4238 27492475 PMC5066309

[124] Qian B-Z Pollard JW. Macrophage Diversity Enhances Tumor Progression and Metastasis. Cell (2010) 141(1):39–51. doi: 10.1016/j.cell.2010.03.014 20371344 PMC4994190

[125] Kumar V Cheng P Condamine T Mony S Languino LR McCaffrey JC. CD45 Phosphatase Inhibits STAT3 Transcription Factor Activity in Myeloid Cells and Promotes Tumor-Associated Macrophage Differentiation. Immunity (2016) 44(2):303–15. doi: 10.1016/j.immuni.2016.01.014 PMC4759655 26885857

[126] Bronte V Brandau S Chen S-H Colombo MP Frey AB Greten TF. Recommendations for Myeloid-Derived Suppressor Cell Nomenclature and Characterization Standards. Nat Commun (2016) 7(1):12150. doi: 10.1038/ncomms12150 27381735 PMC4935811

[127] Bottazzi B Erba E Nobili N Fazioli F Rambaldi A Mantovani A. A Paracrine Circuit in the Regulation of the Proliferation of Macrophages Infiltrating Murine Sarcomas. J Immunol (1990) 144(6):2409–12. 2138198

[128] Campbell MJ Tonlaar NY Garwood ER Huo D Moore DH Khramtsov AI. Proliferating Macrophages Associated With High Grade, Hormone Receptor Negative Breast Cancer and Poor Clinical Outcome. Breast Cancer Res Treat (2011) 128(3):703–11. doi: 10.1007/s10549-010-1154-y PMC4657137 20842526

[129] Movahedi K Laoui D Gysemans C Baeten M Stangé G den BJV. Different Tumor Microenvironments Contain Functionally Distinct Subsets of Macrophages Derived From Ly6C(high) Monocytes. Cancer Res (2010) 70(14):5728–39. doi: 10.1158/0008-5472.CAN-09-4672 20570887

[130] Pittet MJ Michielin O Migliorini D. Clinical Relevance of Tumour-Associated Macrophages. Nat Rev Clin Oncol (2022), 1–20. doi: 10.1038/s41571-022-00620-6 35354979

[131] Chen Z Feng X Herting CJ Garcia VA Nie K Pong WW. Cellular and Molecular Identity of Tumor-Associated Macrophages in Glioblastoma. Cancer Res (2017) 77(9):2266–78. doi: 10.1158/0008-5472.CAN-16-2310 PMC5741820 28235764

[132] DeNardo DG Barreto JB Andreu P Vasquez L Tawfik D Kolhatkar N. CD4(+) T Cells Regulate Pulmonary Metastasis of Mammary Carcinomas by Enhancing Protumor Properties of Macrophages. Cancer Cell (2009) 16(2):91–102. doi: 10.1016/j.ccr.2009.06.018 19647220 PMC2778576

[133] Evans R Alexander P. Cooperation of Immune Lymphoid Cells With Macrophages in Tumour Immunity. Nature (1970) 228(5272):620–2. doi: 10.1038/228620a0 5529055

[134] Mantovani A Bottazzi B Colotta F Sozzani S Ruco L. The Origin and Function of Tumor-Associated Macrophages. Immunol Today (1992) 13(7):265–70. doi: 10.1016/0167-5699(92)90008-U 1388654

[135] Adams DO Hamilton TA. The Cell Biology of Macrophage Activation. Annu Rev Immunol (1984) 2:283–318. doi: 10.1146/annurev.iy.02.040184.001435 6100475

[136] Prehn RT. The Immune Reaction as a Stimulator of Tumor Growth. Science (1972) 176(4031):170–1. doi: 10.1126/science.176.4031.170 5014438

[137] Zhu Y Herndon JM Sojka DK Kim K-W Knolhoff BL Zuo C. Tissue-Resident Macrophages in Pancreatic Ductal Adenocarcinoma Originate From Embryonic Hematopoiesis and Promote Tumor Progression. Immunity (2017) 47(2):323–338.e6. doi: 10.1016/j.immuni.2017.07.014 28813661 PMC5578409

[138] Lin EY Nguyen AV Russell RG Pollard JW. Colony-Stimulating Factor 1 Promotes Progression of Mammary Tumors to Malignancy. J Exp Med (2001) 193(6):727–40. doi: 10.1084/jem.193.6.727 PMC2193412 11257139

[139] Hanahan D Coussens LM. Accessories to the Crime: Functions of Cells Recruited to the Tumor Microenvironment. Cancer Cell (2012) 21(3):309–22. doi: 10.1016/j.ccr.2012.02.022 22439926

[140] Pollard JW. Trophic Macrophages in Development and Disease. Nat Rev Immunol (2009) 9(4):259–70. doi: 10.1038/nri2528 PMC3648866 19282852

[141] Helm O Held-Feindt J Grage-Griebenow E Reiling N Ungefroren H Vogel I. Tumor-Associated Macrophages Exhibit Pro- and Anti-Inflammatory Properties by Which They Impact on Pancreatic Tumorigenesis. Int J Cancer (2014) 135(4):843–61. doi: 10.1002/ijc.28736 24458546

[142] Lewis JS Landers RJ Underwood JC Harris AL Lewis CE. Expression of Vascular Endothelial Growth Factor by Macrophages Is Up-Regulated in Poorly Vascularized Areas of Breast Carcinomas. J Pathol (2000) 192(2):150–8. doi: 10.1002/1096-9896(2000)9999:9999<::AID-PATH687>3.0.CO;2-G 11004690

[143] Stockmann C Doedens A Weidemann A Zhang N Takeda N Greenberg JI. Deletion of Vascular Endothelial Growth Factor in Myeloid Cells Accelerates Tumorigenesis. Nature (2008) 456(7223):814–8. doi: 10.1038/nature07445 PMC3103772 18997773

[144] Leek RD Lewis CE Whitehouse R Greenall M Clarke J Harris AL. Association of Macrophage Infiltration With Angiogenesis and Prognosis in Invasive Breast Carcinoma. Cancer Res (1996) 56(20):4625–9. 8840975

[145] Franklin RA Liao W Sarkar A Kim MV Bivona MR Liu K. The Cellular and Molecular Origin of Tumor-Associated Macrophages. Science (2014) 344(6186):921–5. doi: 10.1126/science.1252510 PMC4204732 24812208

[146] Qian B-Z Li J Zhang H Kitamura T Zhang J Campion LR. CCL2 Recruits Inflammatory Monocytes to Facilitate Breast-Tumour Metastasis. Nature (2011) 475(7355):222–5. doi: 10.1038/nature10138 PMC3208506 21654748

[147] Hanna RN Cekic C Sag D Tacke R Thomas GD Nowyhed H. Patrolling Monocytes Control Tumor Metastasis to the Lung. Science (2015) 350(6263):985–90. doi: 10.1126/science.aac9407 PMC4869713 26494174

[148] Gordon SR Maute RL Dulken BW Hutter G George BM McCracken MN. PD-1 Expression by Tumour-Associated Macrophages Inhibits Phagocytosis and Tumour Immunity. Nature (2017) 545(7655):495–9. doi: 10.1038/nature22396 PMC5931375 28514441

[149] Joyce JA Pollard JW. Microenvironmental Regulation of Metastasis. Nat Rev Cancer (2009) 9(4):239–52. doi: 10.1038/nrc2618 PMC3251309 19279573

[150] Guo X Zhao Y Yan H Yang Y Shen S Dai X. Single Tumor-Initiating Cells Evade Immune Clearance by Recruiting Type II Macrophages. Genes Dev (2017) 31(3):247–59. doi: 10.1101/gad.294348.116 PMC5358722 28223311

[151] Mazzoni M Mauro G Erreni M Romeo P Minna E Vizioli MG. Senescent Thyrocytes and Thyroid Tumor Cells Induce M2-Like Macrophage Polarization of Human Monocytes *via* a PGE2-Dependent Mechanism. J Exp Clin Cancer Res (2019) 38(1):208. doi: 10.1186/s13046-019-1198-8 31113465 PMC6528237

[152] Porta C Consonni FM Morlacchi S Sangaletti S Bleve A Totaro MG. Tumor-Derived Prostaglandin E2 Promotes P50 NF-κb-Dependent Differentiation of Monocytic MDSCs. Cancer Res (2020) 80(13):2874–88. doi: 10.1158/0008-5472.CAN-19-2843 32265223

[153] Pan W Zhu S Qu K Meeth K Cheng J He K. The DNA Methylcytosine Dioxygenase Tet2 Sustains Immunosuppressive Function of Tumor-Infiltrating Myeloid Cells to Promote Melanoma Progression. Immunity (2017) 47(2):284–297.e5. doi: 10.1016/j.immuni.2017.07.020 28813659 PMC5710009

[154] Kryczek I Zou L Rodriguez P Zhu G Wei S Mottram P. B7-H4 Expression Identifies a Novel Suppressive Macrophage Population in Human Ovarian Carcinoma. J Exp Med (2006) 203(4):871–81. doi: 10.1084/jem.20050930 PMC2118300 16606666

[155] Wang L Rubinstein R Lines JL Wasiuk A Ahonen C Guo Y. VISTA, a Novel Mouse Ig Superfamily Ligand That Negatively Regulates T Cell Responses. J Exp Med (2011) 208(3):577–92. doi: 10.1084/jem.20100619 PMC3058578 21383057

[156] Ruffell B Affara NI Coussens LM. Differential Macrophage Programming in the Tumor Microenvironment. Trends Immunol (2012) 33(3):119–26. doi: 10.1016/j.it.2011.12.001 PMC3294003 22277903

[157] Linde N Casanova-Acebes M Sosa MS Mortha A Rahman A Farias E. Macrophages Orchestrate Breast Cancer Early Dissemination and Metastasis. Nat Commun (2018) 9(1):21. doi: 10.1038/s41467-017-02481-5 29295986 PMC5750231

[158] Wang W Liu Y Guo J He H Mi X Chen C. miR-100 Maintains Phenotype of Tumor-Associated Macrophages by Targeting mTOR to Promote Tumor Metastasis *via* Stat5a/IL-1ra Pathway in Mouse Breast Cancer. Oncogenesis (2018) 7(12):1–17. doi: 10.1038/s41389-018-0106-y 30563983 PMC6299090

[159] Costa-Silva B Aiello NM Ocean AJ Singh S Zhang H Thakur BK. Pancreatic Cancer Exosomes Initiate Pre-Metastatic Niche Formation in the Liver. Nat Cell Biol (2015) 17(6):816–26. doi: 10.1038/ncb3169 PMC5769922 25985394

[160] Yin Z Ma T Huang B Lin L Zhou Y Yan J. Macrophage-Derived Exosomal microRNA-501-3p Promotes Progression of Pancreatic Ductal Adenocarcinoma Through the TGFBR3-Mediated TGF-β Signaling Pathway. J Exp Clin Cancer Res (2019) 38(1):310. doi: 10.1186/s13046-019-1313-x 31307515 PMC6631643

[161] Lan J Sun L Xu F Liu L Hu F Song D. M2 Macrophage-Derived Exosomes Promote Cell Migration and Invasion in Colon Cancer. Cancer Res (2019) 79(1):146–58. doi: 10.1158/0008-5472.CAN-18-0014 30401711

[162] Peinado H Alečković M Lavotshkin S Matei I Costa-Silva B Moreno-Bueno G. Melanoma Exosomes Educate Bone Marrow Progenitor Cells Toward a Pro-Metastatic Phenotype Through MET. Nat Med (2012) 18(6):883–91. doi: 10.1038/nm.2753 PMC3645291 22635005

[163] Kitamura T Qian B-Z Soong D Cassetta L Noy R Sugano G. CCL2-Induced Chemokine Cascade Promotes Breast Cancer Metastasis by Enhancing Retention of Metastasis-Associated Macrophages. J Exp Med (2015) 212(7):1043–59. doi: 10.1084/jem.20141836 PMC4493415 26056232

[164] van Deventer HW Palmieri DA Wu QP McCook EC Serody JS. Circulating Fibrocytes Prepare the Lung for Cancer Metastasis by Recruiting Ly-6c+ Monocytes *Via* Ccl2. J Immunol (2013) 190(9):4861–7. doi: 10.4049/jimmunol.1202857 PMC3740355 23536638

[165] Nosaka T Baba T Tanabe Y Sasaki S Nishimura T Imamura Y. Alveolar Macrophages Drive Hepatocellular Carcinoma Lung Metastasis by Generating Leukotriene B4. J Immunol (2018) 200(5):1839–52. doi: 10.4049/jimmunol.1700544 29378914

[166] Sharma SK Chintala NK Vadrevu SK Patel J Karbowniczek M Markiewski MM. Pulmonary Alveolar Macrophages Contribute to the Premetastatic Niche by Suppressing Antitumor T Cell Responses in the Lungs. J Immunol (2015) 194(11):5529–38. doi: 10.4049/jimmunol.1403215 25911761

[167] Kimura Y Inoue A Hangai S Saijo S Negishi H Nishio J. The Innate Immune Receptor Dectin-2 Mediates the Phagocytosis of Cancer Cells by Kupffer Cells for the Suppression of Liver Metastasis. Proc Natl Acad Sci USA (2016) 113(49):14097–102. doi: 10.1073/pnas.1617903113 PMC5150405 27872290

[168] Hambardzumyan D Gutmann DH Kettenmann H. The Role of Microglia and Macrophages in Glioma Maintenance and Progression. Nat Neurosci (2016) 19(1):20–7. doi: 10.1038/nn.4185 PMC4876023 26713745

[169] Pyonteck SM Akkari L Schuhmacher AJ Bowman RL Sevenich L Quail DF. CSF-1R Inhibition Alters Macrophage Polarization and Blocks Glioma Progression. Nat Med (2013) 19(10):1264–72. doi: 10.1038/nm.3337 PMC3840724 24056773

[170] Quail DF Bowman RL Akkari L Quick ML Schuhmacher AJ Huse JT. The Tumor Microenvironment Underlies Acquired Resistance to CSF-1R Inhibition in Gliomas. Science (2016) 352(6288):aad3018. doi: 10.1126/science.aad3018 27199435 PMC5450629

[171] Qiao S Qian Y Xu G Luo Q Zhang Z. Long-Term Characterization of Activated Microglia/Macrophages Facilitating the Development of Experimental Brain Metastasis Through Intravital Microscopic Imaging. J Neuroinflammation (2019) 16(1):4. doi: 10.1186/s12974-018-1389-9 30616691 PMC6323850

[172] Klemm F Maas RR Bowman RL Kornete M Soukup K Nassiri S. Interrogation of the Microenvironmental Landscape in Brain Tumors Reveals Disease-Specific Alterations of Immune Cells. Cell (2020) 181(7):1643–1660.e17. doi: 10.1016/j.cell.2020.05.007 32470396 PMC8558904

[173] Colotta F Peri G Villa A Mantovani A. Rapid Killing of Actinomycin D-Treated Tumor Cells by Human Mononuclear Cells. I. Effectors Belong to the Monocyte-Macrophage Lineage. J Immunol (1984) 132(2):936–44. 6690624

[174] Mantovani A Polentarutti N Luini W Peri G Spreafico F. Role of Host Defense Mechanisms in the Antitumor Activity of Adriamycin and Daunomycin in Mice2. JNCI: J Natl Cancer Inst (1979) 63(1):61–6. 286835

[175] Ma Y Galluzzi L Zitvogel L Kroemer G. Autophagy and Cellular Immune Responses. Immunity (2013) 39(2):211–27. doi: 10.1016/j.immuni.2013.07.017 23973220

[176] Galluzzi L Buqué A Kepp O Zitvogel L Kroemer G. Immunological Effects of Conventional Chemotherapy and Targeted Anticancer Agents. Cancer Cell (2015) 28(6):690–714. doi: 10.1016/j.ccell.2015.10.012 26678337

[177] Pfirschke C Engblom C Rickelt S Cortez-Retamozo V Garris C Pucci F. Immunogenic Chemotherapy Sensitizes Tumors to Checkpoint Blockade Therapy. Immunity (2016) 44(2):343–54. doi: 10.1016/j.immuni.2015.11.024 PMC4758865 26872698

[178] D’Incalci M Badri N Galmarini CM Allavena P. Trabectedin, a Drug Acting on Both Cancer Cells and the Tumour Microenvironment. Br J Cancer (2014) 111(4):646–50. doi: 10.1038/bjc.2014.149 PMC4134488 24755886

[179] Germano G Frapolli R Belgiovine C Anselmo A Pesce S Liguori M. Role of Macrophage Targeting in the Antitumor Activity of Trabectedin. Cancer Cell (2013) 23(2):249–62. doi: 10.1016/j.ccr.2013.01.008 23410977

[180] Germano G Frapolli R Simone M Tavecchio M Erba E Pesce S. Antitumor and Anti-Inflammatory Effects of Trabectedin on Human Myxoid Liposarcoma Cells. Cancer Res (2010) 70(6):2235–44. doi: 10.1158/0008-5472.CAN-09-2335 20215499

[181] Dijkgraaf EM Heusinkveld M Tummers B Vogelpoel LTC Goedemans R Jha V. Chemotherapy Alters Monocyte Differentiation to Favor Generation of Cancer-Supporting M2 Macrophages in the Tumor Microenvironment. Cancer Res (2013) 73(8):2480–92. doi: 10.1158/0008-5472.CAN-12-3542 23436796

[182] Bruchard M Mignot G Derangère V Chalmin F Chevriaux A Végran F. Chemotherapy-Triggered Cathepsin B Release in Myeloid-Derived Suppressor Cells Activates the Nlrp3 Inflammasome and Promotes Tumor Growth. Nat Med (2013) 19(1):57–64. doi: 10.1038/nm.2999 23202296

[183] Jinushi M Chiba S Yoshiyama H Masutomi K Kinoshita I Dosaka-Akita H. Tumor-Associated Macrophages Regulate Tumorigenicity and Anticancer Drug Responses of Cancer Stem/Initiating Cells. PNAS (2011) 108(30):12425–30. doi: 10.1073/pnas.1106645108 PMC3145680 21746895

[184] Hughes R Qian B-Z Rowan C Muthana M Keklikoglou I Olson OC. Perivascular M2 Macrophages Stimulate Tumor Relapse After Chemotherapy. Cancer Res (2015) 75(17):3479–91. doi: 10.1158/0008-5472.CAN-14-3587 PMC5024531 26269531

[185] Mitchem JB Brennan DJ Knolhoff BL Belt BA Zhu Y Sanford DE. Targeting Tumor-Infiltrating Macrophages Decreases Tumor-Initiating Cells, Relieves Immunosuppression, and Improves Chemotherapeutic Responses. Cancer Res (2013) 73(3):1128–41. doi: 10.1158/0008-5472.CAN-12-2731 PMC3563931 23221383

[186] Kridel R Xerri L Gelas-Dore B Tan K Feugier P Vawda A. The Prognostic Impact of CD163-Positive Macrophages in Follicular Lymphoma: A Study From the BC Cancer Agency and the Lymphoma Study Association. Clin Cancer Res (2015) 21(15):3428–35. doi: 10.1158/1078-0432.CCR-14-3253 25869385

[187] Kroemer G Galluzzi L Kepp O Zitvogel L. Immunogenic Cell Death in Cancer Therapy. Annu Rev Immunol (2013) 31(1):51–72. doi: 10.1146/annurev-immunol-032712-100008 23157435

[188] Galluzzi L Buqué A Kepp O Zitvogel L Kroemer G. Immunogenic Cell Death in Cancer and Infectious Disease. Nat Rev Immunol (2017) 17(2):97–111. doi: 10.1038/nri.2016.107 27748397

[189] Di Caro G Cortese N Castino GF Grizzi F Gavazzi F Ridolfi C. Dual Prognostic Significance of Tumour-Associated Macrophages in Human Pancreatic Adenocarcinoma Treated or Untreated With Chemotherapy. Gut (2016) 65(10):1710–20. doi: 10.1136/gutjnl-2015-309193 26156960

[190] Malesci A Bianchi P Celesti G Basso G Marchesi F Grizzi F. Tumor-Associated Macrophages and Response to 5-Fluorouracil Adjuvant Therapy in Stage III Colorectal Cancer. Oncoimmunology (2017) 6(12):e1342918. doi: 10.1080/2162402X.2017.1342918 29209561 PMC5706531

[191] Kodumudi KN Woan K Gilvary DL Sahakian E Wei S Djeu JY. A Novel Chemoimmunomodulating Property of Docetaxel: Suppression of Myeloid-Derived Suppressor Cells in Tumor Bearers. Clin Cancer Res (2010) 16(18):4583–94. doi: 10.1158/1078-0432.CCR-10-0733 PMC3874864 20702612

[192] Mantovani A Allavena P. The Interaction of Anticancer Therapies With Tumor-Associated Macrophages. J Exp Med (2015) 212(4):435–45. doi: 10.1084/jem.20150295 PMC4387285 25753580

[193] Paulus P Stanley ER Schäfer R Abraham D Aharinejad S. Colony-Stimulating Factor-1 Antibody Reverses Chemoresistance in Human MCF-7 Breast Cancer Xenografts. Cancer Res (2006) 66(8):4349–56. doi: 10.1158/0008-5472.CAN-05-3523 16618760

[194] Salvagno C Ciampricotti M Tuit S Hau C-S van Weverwijk A Coffelt SB. Therapeutic Targeting of Macrophages Enhances Chemotherapy Efficacy by Unleashing Type I Interferon Response. Nat Cell Biol (2019) 21(4):511–21. doi: 10.1038/s41556-019-0298-1 PMC6451630 30886344

[195] Neubert NJ Schmittnaegel M Bordry N Nassiri S Wald N Martignier C. T Cell-Induced CSF1 Promotes Melanoma Resistance to PD1 Blockade. Sci Transl Med (2018) 10(436):eaan3311. doi: 10.1126/scitranslmed.aan3311 29643229 PMC5957531

[196] Ho WJ Jaffee EM. Macrophage-Targeting by CSF1/1R Blockade in Pancreatic Cancers. Cancer Res (2021) 81(24):6071–3. doi: 10.1158/0008-5472.CAN-21-3603 PMC9164148 34911778

[197] Yin Y Yao S Hu Y Feng Y Li M Bian Z. The Immune-Microenvironment Confers Chemoresistance of Colorectal Cancer Through Macrophage-Derived Il6. Clin Cancer Res (2017) 23(23):7375–87. doi: 10.1158/1078-0432.CCR-17-1283 28928161

[198] Shree T Olson OC Elie BT Kester JC Garfall AL Simpson K. Macrophages and Cathepsin Proteases Blunt Chemotherapeutic Response in Breast Cancer. Genes Dev (2011) 25(23):2465–79. doi: 10.1101/gad.180331.111 PMC3243057 22156207

[199] Xu J Escamilla J Mok S David J Priceman S West B. CSF1R Signaling Blockade Stanches Tumor-Infiltrating Myeloid Cells and Improves the Efficacy of Radiotherapy in Prostate Cancer. Cancer Res (2013) 73(9):2782–94. doi: 10.1158/0008-5472.CAN-12-3981 PMC4097014 23418320

[200] Durante M Reppingen N Held KD. Immunologically Augmented Cancer Treatment Using Modern Radiotherapy. Trends Mol Med (2013) 19(9):565–82. doi: 10.1016/j.molmed.2013.05.007 23831337

[201] Klug F Prakash H Huber PE Seibel T Bender N Halama N. Low-Dose Irradiation Programs Macrophage Differentiation to an iNOS^+^/M1 Phenotype That Orchestrates Effective T Cell Immunotherapy. Cancer Cell (2013) 24(5):589–602. doi: 10.1016/j.ccr.2013.09.014 24209604

[202] Teresa Pinto A Laranjeiro Pinto M Patrícia Cardoso A Monteiro C Teixeira Pinto M Filipe Maia A. Ionizing Radiation Modulates Human Macrophages Towards a Pro-Inflammatory Phenotype Preserving Their Pro-Invasive and Pro-Angiogenic Capacities. Sci Rep (2016) 6:18765. doi: 10.1038/srep18765 26735768 PMC4702523

[203] Niesel K Schulz M Anthes J Alekseeva T Jadranka M Salamero-Boix A. The Immune Suppressive Microenvironment Affects Efficacy of Radio-Immunotherapy in Brain Metastasis. EMBO Mol Med (2021) 13(5):e13412. doi: 10.15252/emmm.202013412 33755340 PMC8103101

[204] Iida N Dzutsev A Stewart CA Smith L Bouladoux N Weingarten RA. Commensal Bacteria Control Cancer Response to Therapy by Modulating the Tumor Microenvironment. Science (2013) 342(6161):967–70. doi: 10.1126/science.1240527 PMC6709532 24264989

[205] Viaud S Saccheri F Mignot G Yamazaki T Daillère R Hannani D. The Intestinal Microbiota Modulates the Anticancer Immune Effects of Cyclophosphamide. Science (2013) 342(6161):971–6. doi: 10.1126/science.1240537 PMC4048947 24264990

[206] Vétizou M Pitt JM Daillère R Lepage P Waldschmitt N Flament C. Anticancer Immunotherapy by CTLA-4 Blockade Relies on the Gut Microbiota. Science (2015) 350(6264):1079–84. doi: 10.1126/science.aad1329 PMC4721659 26541610

[207] Alexander JL Wilson ID Teare J Marchesi JR Nicholson JK Kinross JM. Gut Microbiota Modulation of Chemotherapy Efficacy and Toxicity. Nat Rev Gastroenterol Hepatol (2017) 14(6):356–65. doi: 10.1038/nrgastro.2017.20 28270698

[208] Hayase E Jenq RR. Role of the Intestinal Microbiome and Microbial-Derived Metabolites in Immune Checkpoint Blockade Immunotherapy of Cancer. Genome Med (2021) 13(1):107. doi: 10.1186/s13073-021-00923-w 34162429 PMC8220726

[209] Vacchelli E Ma Y Baracco EE Sistigu A Enot DP Pietrocola F. Chemotherapy-Induced Antitumor Immunity Requires Formyl Peptide Receptor 1. Science (2015) 350(6263):972–8. doi: 10.1126/science.aad0779 26516201

[210] Baracco EE Pietrocola F Buqué A Bloy N Senovilla L Zitvogel L. Inhibition of Formyl Peptide Receptor 1 Reduces the Efficacy of Anticancer Chemotherapy Against Carcinogen-Induced Breast Cancer. Oncoimmunology (2016) 5(6):e1139275. doi: 10.1080/2162402X.2016.1139275 27471610 PMC4938360

[211] Cavnar MJ Zeng S Kim TS Sorenson EC Ocuin LM Balachandran VP. KIT Oncogene Inhibition Drives Intratumoral Macrophage M2 Polarization. J Exp Med (2013) 210(13):2873–86. doi: 10.1084/jem.20130875 PMC3865475 24323358

[212] Sprinzl MF Reisinger F Puschnik A Ringelhan M Ackermann K Hartmann D. Sorafenib Perpetuates Cellular Anticancer Effector Functions by Modulating the Crosstalk Between Macrophages and Natural Killer Cells. Hepatology (2013) 57(6):2358–68. doi: 10.1002/hep.26328 23424039

[213] Qian B-Z Zhang H Li J He T Yeo E-J Soong DYH. FLT1 Signaling in Metastasis-Associated Macrophages Activates an Inflammatory Signature That Promotes Breast Cancer Metastasis. J Exp Med (2015) 212(9):1433–48. doi: 10.1084/jem.20141555 PMC4548055 26261265

[214] Larionova I Tuguzbaeva G Ponomaryova A Stakheyeva M Cherdyntseva N Pavlov V. Tumor-Associated Macrophages in Human Breast, Colorectal, Lung, Ovarian and Prostate Cancers. Front Oncol (2020), 10:566511. doi: 10.3389/fonc.2020.566511 33194645 PMC7642726

[215] Algars A Irjala H Vaittinen S Huhtinen H Sundström J Salmi M. Type and Location of Tumor-Infiltrating Macrophages and Lymphatic Vessels Predict Survival of Colorectal Cancer Patients. Int J Cancer (2012) 131(4):864–73. doi: 10.1002/ijc.26457 21952788

[216] Vankerckhoven A Wouters R Mathivet T Ceusters J Baert T Van Hoylandt A. Opposite Macrophage Polarization in Different Subsets of Ovarian Cancer: Observation From a Pilot Study. Cells (2020) 9(2):305. doi: 10.3390/cells9020305 PMC7072171 32012728

[217] Kloepper J Riedemann L Amoozgar Z Seano G Susek K Yu V. Ang-2/VEGF Bispecific Antibody Reprograms Macrophages and Resident Microglia to Anti-Tumor Phenotype and Prolongs Glioblastoma Survival. PNAS (2016) 113(16):4476–81. doi: 10.1073/pnas.1525360113 PMC4843473 27044098

[218] Jassar AS Suzuki E Kapoor V Sun J Silverberg MB Cheung L. Activation of Tumor-Associated Macrophages by the Vascular Disrupting Agent 5,6-Dimethylxanthenone-4-Acetic Acid Induces an Effective CD8+ T-Cell–Mediated Antitumor Immune Response in Murine Models of Lung Cancer and Mesothelioma. Cancer Res (2005) 65(24):11752–61. doi: 10.1158/0008-5472.CAN-05-1658 16357188

[219] Loke P Allison JP. PD-L1 and PD-L2 are Differentially Regulated by Th1 and Th2 Cells. Proc Natl Acad Sci USA (2003) 100(9):5336–41. doi: 10.1073/pnas.0931259100 PMC154346 12697896

[220] Noman MZ Desantis G Janji B Hasmim M Karray S Dessen P. PD-L1 Is a Novel Direct Target of HIF-1α, and Its Blockade Under Hypoxia Enhanced MDSC-Mediated T Cell Activation. J Exp Med (2014) 211(5):781–90. doi: 10.1084/jem.20131916 PMC4010891 24778419

[221] Relecom A Merhi M Inchakalody V Uddin S Rinchai D Bedognetti D. Emerging Dynamics Pathways of Response and Resistance to PD-1 and CTLA-4 Blockade: Tackling Uncertainty by Confronting Complexity. J Exp Clin Cancer Res (2021) 40(1):74. doi: 10.1186/s13046-021-01872-3 33602280 PMC7893879

[222] Tumeh PC Harview CL Yearley JH Shintaku IP Taylor EJM Robert L. PD-1 Blockade Induces Responses by Inhibiting Adaptive Immune Resistance. Nature (2014) 515(7528):568–71. doi: 10.1038/nature13954 PMC4246418 25428505

[223] Selby MJ Engelhardt JJ Quigley M Henning KA Chen T Srinivasan M. Anti-CTLA-4 Antibodies of IgG2a Isotype Enhance Antitumor Activity Through Reduction of Intratumoral Regulatory T Cells. Cancer Immunol Res (2013) 1(1):32–42. doi: 10.1158/2326-6066.CIR-13-0013 24777248

[224] Stone EL O’Brien EM. Investigating the Effect of Anti-CTLA-4 on Tumor-Infiltrating Effector T Cells. J Immunol (2017) 198(1 Supplement):56.12–2.

[225] Romano E Kusio-Kobialka M Foukas PG Baumgaertner P Meyer C Ballabeni P. Ipilimumab-Dependent Cell-Mediated Cytotoxicity of Regulatory T Cells *Ex Viv*o by Nonclassical Monocytes in Melanoma Patients. Proc Natl Acad Sci USA (2015) 112(19):6140–5. doi: 10.1073/pnas.1417320112 PMC4434760 25918390

[226] Vaddepally RK Kharel P Pandey R Garje R Chandra AB. Review of Indications of FDA-Approved Immune Checkpoint Inhibitors Per NCCN Guidelines With the Level of Evidence. Cancers (Basel) (2020) 12(3):738. doi: 10.3390/cancers12030738 PMC7140028 32245016

[227] Perdiguero EG Geissmann F. The Development and Maintenance of Resident Macrophages. Nat Immunol (2016) 17(1):2–8. doi: 10.1038/ni.3341 26681456 PMC4950995

[228] Lin H Lee E Hestir K Leo C Huang M Bosch E. Discovery of a Cytokine and its Receptor by Functional Screening of the Extracellular Proteome. Science (2008) 320(5877):807–11. doi: 10.1126/science.1154370 18467591

[229] Baghdadi M Wada H Nakanishi S Abe H Han N Putra WE. Chemotherapy-Induced IL34 Enhances Immunosuppression by Tumor-Associated Macrophages and Mediates Survival of Chemoresistant Lung Cancer Cells. Cancer Res (2016) 76(20):6030–42. doi: 10.1158/0008-5472.CAN-16-1170 27550451

[230] Scholl SM Lidereau R de la Rochefordière A Le-Nir CC Mosseri V Noguès C. Circulating Levels of the Macrophage Colony Stimulating Factor CSF-1 in Primary and Metastatic Breast Cancer Patients. Pilot Study Breast Cancer Res Treat (1996) 39(3):275–83. doi: 10.1007/BF01806155 8877007

[231] Kowal J Kornete M Joyce JA. Re-Education of Macrophages as a Therapeutic Strategy in Cancer. Immunotherapy (2019) 11(8):677–89. doi: 10.2217/imt-2018-0156 31088236

[232] Klemm F Möckl A Salamero-Boix A Alekseeva T Schäffer A Schulz M. Compensatory CSF2-Driven Macrophage Activation Promotes Adaptive Resistance to CSF1R Inhibition in Breast-to-Brain Metastasis. Nat Cancer (2021) 2(10):1086–101. doi: 10.1038/s43018-021-00254-0 35121879

[233] Olson OC Kim H Quail DF Foley EA Joyce JA. Tumor-Associated Macrophages Suppress the Cytotoxic Activity of Antimitotic Agents. Cell Rep (2017) 19(1):101–13. doi: 10.1016/j.celrep.2017.03.038 PMC5614506 28380350

[234] Valero JG Matas-Céspedes A Arenas F Rodriguez V Carreras J Serrat N. The Receptor of the Colony-Stimulating Factor-1 (CSF-1R) Is a Novel Prognostic Factor and Therapeutic Target in Follicular Lymphoma. Leukemia (2021) 35(9):2635–49. doi: 10.1038/s41375-021-01201-9 PMC8410584 33731849

[235] Stafford JH Hirai T Deng L Chernikova SB Urata K West BL. Colony Stimulating Factor 1 Receptor Inhibition Delays Recurrence of Glioblastoma After Radiation by Altering Myeloid Cell Recruitment and Polarization. Neuro Oncol (2016) 18(6):797–806. doi: 10.1093/neuonc/nov272 26538619 PMC4864255

[236] Peña CG Nakada Y Saatcioglu HD Aloisio GM Cuevas I Zhang S. LKB1 Loss Promotes Endometrial Cancer Progression *via* CCL2-Dependent Macrophage Recruitment. J Clin Invest (2015) 125(11):4063–76. doi: 10.1172/JCI82152 PMC4639978 26413869

[237] Hao Q Vadgama JV Wang P. CCL2/CCR2 Signaling in Cancer Pathogenesis. Cell Commun Signaling (2020) 18(1):82. doi: 10.1186/s12964-020-00589-8 PMC7257158 32471499

[238] Lebrecht A Grimm C Lantzsch T Ludwig E Hefler L Ulbrich E. Monocyte Chemoattractant Protein-1 Serum Levels in Patients With Breast Cancer. Tumour Biol (2004) 25(1–2):14–7. doi: 10.1159/000077718 15192307

[239] Loberg RD Ying C Craig M Yan L Snyder LA Pienta KJ. CCL2 as an Important Mediator of Prostate Cancer Growth *In Vivo* Through the Regulation of Macrophage Infiltration. Neoplasia (2007) 9(7):556–62. doi: 10.1593/neo.07307 PMC1939930 17710158

[240] Bonapace L Coissieux M-M Wyckoff J Mertz KD Varga Z Junt T. Cessation of CCL2 Inhibition Accelerates Breast Cancer Metastasis by Promoting Angiogenesis. Nature (2014) 515(7525):130–3. doi: 10.1038/nature13862 25337873

[241] Nywening TM Wang-Gillam A Sanford DE Belt BA Panni RZ Cusworth BM. Targeting Tumour-Associated Macrophages With CCR2 Inhibition in Combination With FOLFIRINOX in Patients With Borderline Resectable and Locally Advanced Pancreatic Cancer: A Single-Centre, Open-Label, Dose-Finding, non-Randomised, Phase 1b Trial. Lancet Oncol (2016) 17(5):651–62. doi: 10.1016/S1470-2045(16)00078-4 PMC5407285 27055731

[242] Zhao L Lim SY Gordon-Weeks AN Tapmeier TT Im JH Cao Y. Recruitment of a Myeloid Cell Subset (CD11b/Gr1 Mid) *via* CCL2/CCR2 Promotes the Development of Colorectal Cancer Liver Metastasis. Hepatology (2013) 57(2):829–39. doi: 10.1002/hep.26094 23081697

[243] Feng M Jiang W Kim BYS Zhang CC Fu Y-X Weissman IL. Phagocytosis Checkpoints as New Targets for Cancer Immunotherapy. Nat Rev Cancer (2019) 19(10):568–86. doi: 10.1038/s41568-019-0183-z PMC7002027 31462760

[244] Zhang H Lu H Xiang L Bullen JW Zhang C Samanta D. HIF-1 Regulates CD47 Expression in Breast Cancer Cells to Promote Evasion of Phagocytosis and Maintenance of Cancer Stem Cells. Proc Natl Acad Sci USA (2015) 112(45):E6215–6223. doi: 10.1073/pnas.1520032112 PMC4653179 26512116

[245] Edris B Weiskopf K Volkmer AK Volkmer J-P Willingham SB Contreras-Trujillo H. Antibody Therapy Targeting the CD47 Protein Is Effective in a Model of Aggressive Metastatic Leiomyosarcoma. Proc Natl Acad Sci USA (2012) 109(17):6656–61. doi: 10.1073/pnas.1121629109 PMC3340056 22451919

[246] Blazar BR Lindberg FP Ingulli E Panoskaltsis-Mortari A Oldenborg PA Iizuka K. CD47 (Integrin-Associated Protein) Engagement of Dendritic Cell and Macrophage Counterreceptors Is Required to Prevent the Clearance of Donor Lymphohematopoietic Cells. J Exp Med (2001) 194(4):541–9. doi: 10.1084/jem.194.4.541 PMC2193501 11514609

[247] Wang Y Wang H Bronson R Fu Y Yang Y-G. Rapid Dendritic Cell Activation and Resistance to Allotolerance Induction in Anti-CD154-Treated Mice Receiving CD47-Deficient Donor-Specific Transfusion. Cell Transpl (2014) 23(3):355–63. doi: 10.3727/096368912X661346 PMC3732805 23295133

[248] Gholamin S Mitra SS Feroze AH Liu J Kahn SA Zhang M. Disrupting the CD47-Sirpα Anti-Phagocytic Axis by a Humanized Anti-CD47 Antibody Is an Efficacious Treatment for Malignant Pediatric Brain Tumors. Sci Transl Med (2017) 9(381):eaaf2968. doi: 10.1126/scitranslmed.aaf2968 28298418

[249] Hernandez MGH Shen L Rock KL. CD40 on APCs is Needed for Optimal Programming, Maintenance, and Recall of CD8+ T Cell Memory Even in the Absence of CD4+ T Cell Help. J Immunol (2008) 180(7):4382–90. doi: 10.4049/jimmunol.180.7.4382 18354158

[250] Richards DM Sefrin JP Gieffers C Hill O Merz C. Concepts for Agonistic Targeting of CD40 in Immuno-Oncology. Hum Vaccin Immunother (2020) 16(2):377–87. doi: 10.1080/21645515.2019.1653744 PMC7062441 31403344

[251] Vonderheide RH. CD40 Agonist Antibodies in Cancer Immunotherapy. Annu Rev Med (2020) 71:47–58. doi: 10.1146/annurev-med-062518-045435 31412220

[252] Beatty GL Chiorean EG Fishman MP Saboury B Teitelbaum UR Sun W. CD40 Agonists Alter Tumor Stroma and Show Efficacy Against Pancreatic Carcinoma in Mice and Humans. Science (2011) 331(6024):1612–6. doi: 10.1126/science.1198443 PMC3406187 21436454

[253] Hoves S Ooi C-H Wolter C Sade H Bissinger S Schmittnaegel M. Rapid Activation of Tumor-Associated Macrophages Boosts Preexisting Tumor Immunity. J Exp Med (2018) 215(3):859–76. doi: 10.1084/jem.20171440 PMC5839760 29436396

[254] Rüter J Antonia SJ Burris HA Huhn RD Vonderheide RH. Immune Modulation With Weekly Dosing of an Agonist CD40 Antibody in a Phase I Study of Patients With Advanced Solid Tumors. Cancer Biol Ther (2010) 10(10):983–93. doi: 10.4161/cbt.10.10.13251 PMC3047092 20855968

[255] O’Hara MH O’Reilly EM Varadhachary G Wolff RA Wainberg ZA Ko AH. CD40 Agonistic Monoclonal Antibody APX005M (Sotigalimab) and Chemotherapy, With or Without Nivolumab, for the Treatment of Metastatic Pancreatic Adenocarcinoma: An Open-Label, Multicentre, Phase 1b Study. Lancet Oncol (2021) 22(1):118–31. doi: 10.1016/S1470-2045(20)30532-5 33387490

[256] Kaneda MM Messer KS Ralainirina N Li H Leem CJ Gorjestani S. Pi3kγ Is a Molecular Switch That Controls Immune Suppression. Nature (2016) 539(7629):437–42. doi: 10.1038/nature19834 PMC5479689 27642729

[257] De Henau O Rausch M Winkler D Campesato LF Liu C Cymerman DH. Overcoming Resistance to Checkpoint Blockade Therapy by Targeting PI3Kγ in Myeloid Cells. Nature (2016) 539(7629):443–7. doi: 10.1038/nature20554 PMC5634331 27828943

[258] Deczkowska A Weiner A Amit I. The Physiology, Pathology, and Potential Therapeutic Applications of the TREM2 Signaling Pathway. Cell (2020) 181(6):1207–17. doi: 10.1016/j.cell.2020.05.003 32531244

[259] Molgora M Esaulova E Vermi W Hou J Chen Y Luo J. TREM2 Modulation Remodels the Tumor Myeloid Landscape Enhancing Anti-PD-1 Immunotherapy. Cell (2020) 182(4):886–900.e17. doi: 10.1016/j.cell.2020.07.013 32783918 PMC7485282

[260] Katzenelenbogen Y Sheban F Yalin A Yofe I Svetlichnyy D Jaitin DA. Coupled scRNA-Seq and Intracellular Protein Activity Reveal an Immunosuppressive Role of TREM2 in Cancer. Cell (2020) 182(4):872–885.e19. doi: 10.1016/j.cell.2020.06.032 32783915

[261] Pettenati C Ingersoll MA. Mechanisms of BCG Immunotherapy and Its Outlook for Bladder Cancer. Nat Rev Urol (201) 15(10):615–25. doi: 10.1038/s41585-018-0055-4 29991725

[262] Ji N Mukherjee N Morales EE Tomasini ME Hurez V Curiel TJ. Percutaneous BCG Enhances Innate Effector Antitumor Cytotoxicity During Treatment of Bladder Cancer: A Translational Clinical Trial. Oncoimmunology (2019) 8(8):1614857. doi: 10.1080/2162402X.2019.1614857 31413921 PMC6682354

[263] Fitzgerald KA Kagan JC. Toll-Like Receptors and the Control of Immunity. Cell (2020) 180(6):1044–66. doi: 10.1016/j.cell.2020.02.041 PMC9358771 32164908

[264] Smith M García-Martínez E Pitter MR Fucikova J Spisek R Zitvogel L. Trial Watch: Toll-Like Receptor Agonists in Cancer Immunotherapy. Oncoimmunology (2018) 7(12):e1526250. doi: 10.1080/2162402X.2018.1526250 30524908 PMC6279325

[265] Kyi C Roudko V Sabado R Saenger Y Loging W Mandeli J. Therapeutic Immune Modulation Against Solid Cancers With Intratumoral Poly-ICLC: A Pilot Trial. Clin Cancer Res (2018) 24(20):4937–48. doi: 10.1158/1078-0432.CCR-17-1866 PMC6191332 29950349

[266] Huang L Xu H Peng G. TLR-Mediated Metabolic Reprogramming in the Tumor Microenvironment: Potential Novel Strategies for Cancer Immunotherapy. Cell Mol Immunol (2018) 15(5):428–37. doi: 10.1038/cmi.2018.4 PMC6068099 29553135

[267] Rodell CB Arlauckas SP Cuccarese MF Garris CS Li R Ahmed MS. TLR7/8-Agonist-Loaded Nanoparticles Promote the Polarization of Tumour-Associated Macrophages to Enhance Cancer Immunotherapy. Nat BioMed Eng (2018) 2(8):578–88. doi: 10.1038/s41551-018-0236-8 PMC6192054 31015631

[268] Anfray C Mainini F Digifico E Maeda A Sironi M Erreni M. Intratumoral Combination Therapy With Poly(I:C) and Resiquimod Synergistically Triggers Tumor-Associated Macrophages for Effective Systemic Antitumoral Immunity. J Immunother Cancer (2021) 9(9):e002408. doi: 10.1136/jitc-2021-002408 34531246 PMC8449972

[269] Singh M Khong H Dai Z Huang X-F Wargo JA Cooper ZA. Effective Innate and Adaptive Antimelanoma Immunity Through Localized TLR7/8 Activation. J Immunol (2014) 193(9):4722–31. doi: 10.4049/jimmunol.1401160 PMC4201984 25252955

[270] Mullins SR Vasilakos JP Deschler K Grigsby I Gillis P John J. Intratumoral Immunotherapy With TLR7/8 Agonist MEDI9197 Modulates the Tumor Microenvironment Leading to Enhanced Activity When Combined With Other Immunotherapies. J ImmunoTher Cancer (2019) 7(1):244. doi: 10.1186/s40425-019-0724-8 31511088 PMC6739946

[271] Frank MJ Reagan PM Bartlett NL Gordon LI Friedberg JW Czerwinski DK. *In Situ* Vaccination With a TLR9 Agonist and Local Low-Dose Radiation Induces Systemic Responses in Untreated Indolent Lymphoma. Cancer Discov (2018) 8(10):1258–69. doi: 10.1158/2159-8290.CD-18-0743 PMC6171524 30154192

[272] Ribas A Medina T Kummar S Amin A Kalbasi A Drabick JJ. SD-101 in Combination With Pembrolizumab in Advanced Melanoma: Results of a Phase Ib, Multicenter Study. Cancer Discov (2018) 8(10):1250–7. doi: 10.1158/2159-8290.CD-18-0280 PMC6719557 30154193

[273] Chow LQM Morishima C Eaton KD Baik CS Goulart BH Anderson LN. Phase Ib Trial of the Toll-Like Receptor 8 Agonist, Motolimod (VTX-2337), Combined With Cetuximab in Patients With Recurrent or Metastatic SCCHN. Clin Cancer Res (2017) 23(10):2442–50. doi: 10.1158/1078-0432.CCR-16-1934 27810904

[274] Viola A Munari F Sánchez-Rodríguez R Scolaro T Castegna A. The Metabolic Signature of Macrophage Responses. Front Immunol (2019) 10:1462. doi: 10.3389/fimmu.2019.01462 PMC6618143 31333642

[275] Oh M-H Sun I-H Zhao L Leone RD Sun I-M Xu W. Targeting Glutamine Metabolism Enhances Tumor-Specific Immunity by Modulating Suppressive Myeloid Cells. J Clin Invest (2020) 130(7):3865–84. doi: 10.1172/JCI131859 PMC7324212 32324593

[276] Menga A Serra M Todisco S Riera-Domingo C Ammarah U Ehling M. Glufosinate Constrains Synchronous and Metachronous Metastasis by Promoting Anti-Tumor Macrophages. EMBO Mol Med (2020) 12(10):e11210. doi: 10.15252/emmm.201911210 32885605 PMC7539200

[277] Manoharan I Prasad PD Thangaraju M Manicassamy S. Lactate-Dependent Regulation of Immune Responses by Dendritic Cells and Macrophages. Front Immunol (2021) 12:691134. doi: 10.3389/fimmu.2021.691134 34394085 PMC8358770

[278] Chen P Zuo H Xiong H Kolar MJ Chu Q Saghatelian A. Gpr132 Sensing of Lactate Mediates Tumor-Macrophage Interplay to Promote Breast Cancer Metastasis. Proc Natl Acad Sci USA (2017) 114(3):580–5. doi: 10.1073/pnas.1614035114 PMC5255630 28049847

[279] Mu X Shi W Xu Y Xu C Zhao T Geng B. Tumor-Derived Lactate Induces M2 Macrophage Polarization *via* the Activation of the ERK/STAT3 Signaling Pathway in Breast Cancer. Cell Cycle (2018) 17(4):428–38. doi: 10.1080/15384101.2018.1444305 PMC5927648 29468929

[280] Geeraerts X Bolli E Fendt S-M Van Ginderachter JA. Macrophage Metabolism As Therapeutic Target for Cancer, Atherosclerosis, and Obesity. Front Immunol (2017) 8:289. doi: 10.3389/fimmu.2017.00289 28360914 PMC5350105

[281] Vigano S Alatzoglou D Irving M Ménétrier-Caux C Caux C Romero P. Targeting Adenosine in Cancer Immunotherapy to Enhance T-Cell Function. Front Immunol (2019), 10:925. doi: 10.3389/fimmu.2019.00925 31244820 PMC6562565

[282] Cekic C Day Y-J Sag D Linden J. Myeloid Expression of Adenosine A2A Receptor Suppresses T and NK Cell Responses in the Solid Tumor Microenvironment. Cancer Res (2014) 74(24):7250–9. doi: 10.1158/0008-5472.CAN-13-3583 PMC4459782 25377469

[283] Ma S-R Deng W-W Liu J-F Mao L Yu G-T Bu L-L. Blockade of Adenosine A2A Receptor Enhances CD8+ T Cells Response and Decreases Regulatory T Cells in Head and Neck Squamous Cell Carcinoma. Mol Cancer (2017) 16(1):99. doi: 10.1186/s12943-017-0665-0 28592285 PMC5461710

[284] Guerriero JL Sotayo A Ponichtera HE Castrillon JA Pourzia AL Schad S. Class IIa HDAC Inhibition Reduces Breast Tumours and Metastases Through Anti-Tumour Macrophages. Nature (2017) 543(7645):428–32. doi: 10.1038/nature21409 PMC8170529 28273064

[285] Lu Z Zou J Li S Topper MJ Tao Y Zhang H. Epigenetic Therapy Inhibits Metastases by Disrupting Premetastatic Niches. Nature (2020) 579(7798):284–90. doi: 10.1038/s41586-020-2054-x PMC8765085 32103175

[286] Frigault MJ Maus MV. State of the Art in CAR T Cell Therapy for CD19^+^ B Cell Malignancies. J Clin Invest (2020) 130(4):1586–94. doi: 10.1172/JCI129208 PMC7108913 32235098

[287] Klichinsky M Ruella M Shestova O Lu XM Best A Zeeman M. Human Chimeric Antigen Receptor Macrophages for Cancer Immunotherapy. Nat Biotechnol (2020) 38(8):947–53. doi: 10.1038/s41587-020-0462-y PMC7883632 32361713

[288] Schmid MC Avraamides CJ Dippold HC Franco I Foubert P Ellies LG. Receptor Tyrosine Kinases and TLR/IL1Rs Unexpectedly Activate Myeloid Cell Pi3kγ, A Single Convergent Point Promoting Tumor Inflammation and Progression. Cancer Cell (2011) 19(6):715–27. doi: 10.1016/j.ccr.2011.04.016 PMC3144144 21665146

